# Electronic Manifestations of Scandide Contraction: Theoretical Photoelectron Spectroscopy of Monovalent Group 13 Compounds

**DOI:** 10.1021/acs.inorgchem.5c02000

**Published:** 2025-07-12

**Authors:** Jeanet Conradie, Kristian Torstensen, Pekka Pyykkö, Abhik Ghosh

**Affiliations:** † Department of Chemistry, 8016 UiT—The Arctic University of Norway, N-9037 Tromsø, Norway; ‡ Department of Chemistry, 37702 University of the Free State, P.O. Box 339, Bloemfontein 9300, Republic of South Africa; § Department of Chemistry, Faculty of Science, University of Helsinki, POB 55, 00014 Helsinki, Finland

## Abstract

A wide-ranging density functional theory (DFT) survey of monovalent Group 13 complexes (trielylenes) has afforded detailed insights into periodic trends in these compounds. Five classes of neutral complexes were examined, based on β-diketiminate, bis­(imino)­carbazolate (pincer), hydrotrispyrazolylborate, cyclopentadienide, and monocoordinate aryl ligands. Also examined was a set of *N*,*N*′-diaryl-1,4-diazabutadiene dianion-coordinated triel­(I) anions. In general, the ionization potential of the Ga-based lone pairs was found to be nearly 1 eV or more higher than that of Al-based lone pairs in similar species; the corresponding IPs for In were found to hover around the values calculated for Ga. This general trend may be thought of as an electronic manifestation of scandide contraction, which results in stabilization of the 4s and 4p subshells due to poor screening by the filled 3d subshell. Only for the aryl series was this effect found to be quite muted, apparently because these complexes do not harbor a genuine metal-based lone pair. Instead, the highest occupied molecular orbital (HOMO) consists of a metal–carbon σ-antibonding orbital, spread over multiple atoms. The calculations also underscore the major role of the supporting ligand in modulating the electronic properties of trielylenes and help explain why certain species such as Al­(I) bis­(imino)­carbazolates might not be stable enough to permit isolation.

## Graphical abstract

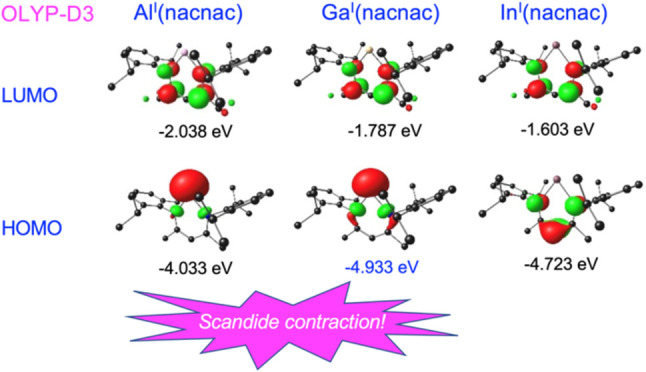

## Introduction

Over the last few decades, the chemistry of low-oxidation-state Group 13 (triel) compounds has grown into a vibrant subfield of modern main group chemistry.^1^ For most of the twentieth century, the area was dominated by small matrix-isolated species and cluster compounds.^2–6^ Some of the first reports of fully structurally characterized, mononuclear, monovalent aluminum and gallium complexes came in the year 2000.^7,8^ Today, a wide variety of such complexes are available, as are excellent review articles dedicated to the subject.^9–15^ Notably absent, however, is a comprehensive quantum chemical survey of the field, highlighting periodic trends across different classes of compounds. In this study, we have attempted to address this lacuna, focusing on potentially observable electronic properties of five classes of monovalent compounds of the Al, Ga, and In triad.^16^

Before proceeding further, it may be useful to emphasize the difference between the terms valence, oxidation number, coordination number, and formal charge. For the species examined in this study, valence and oxidation number are generally the same, but the two quantities differ in metal–metal-bonded molecules and in cluster compounds (since bonds between atoms of the same element contribute to valence but not toward oxidation number). We refer to Parkin’s excellent article for a full discussion,^17^ but would simply like to draw attention to the equation “valence = no. of bonds + formal charge” so the reader may apply it to any given atom in a structural formula to determine its valence state. With that background, monovalent triel species may be viewed as analogues of singlet carbenes^10^ and, by analogy with tetrylenes,^18–20^ may be referred to as triylenes or trielylenes. (To avoid confusion with triyls or triradicals, we have used the latter term in this work). Like singlet carbenes, trielylenes are ambiphilic species, exhibiting both nucleophilic and electrophilic character. Given the detailed insights that gas-phase photoelectron spectroscopy (including negative ion photoelectron spectroscopy^21^) has afforded relative to the electronic structure of carbenes,^22^ we have been intrigued by the prospect of similar measurements on trielylenes—a proposition that is easier envisioned than accomplished. Key impediments include not only the sensitive nature of the compounds, but also the need for relatively large quantities of samples and specialized, custom-built equipment.

Fortunately, a simple workaround was at hand. Over the years, we realized that density functional theory (DFT) calculations provide quick access to accurate vertical and adiabatic ionization potentials for a wide range of molecules, including carbenes,^23^ silylenes,^24,25^ strained hydrocarbons,^26^ fullerenes,^27–29^ porphyrins and related compounds,^30–34^ and metal–metal multiple-bonded complexes.^35,36^ The agreement with experimental ionization potentials and electron affinities is generally excellent, often to within 0.1–0.2 eV. Armed with this insight, we embarked on a theoretical photoelectron spectroscopic study of a substantial range of monovalent Group 13 complexes involving aluminum, gallium and indium. The results underscored the critical role of the ligand in determining key electronic properties of the molecules such as ionization potentials, electron affinities, and singlet–triplet gaps.

Gratifyingly, the results also yielded significant insights into periodic trends for low-valent triel species, which may be briefly contextualized as follows. With radially nodeless 2p orbitals, the period-2 elements B–Ne are atypical and exhibit some of the most extreme chemical properties (such as the extreme electronegativity of fluorine).^37,38^ With radial nodes in both 3s and 3p orbitals, the period-3 elements Al–Ar may be viewed as normal main-group elements. The period-4 elements Ga–Kr incorporate a new feature, a poorly screening, filled 3d subshell, which results in unexpectedly short ionic and single-bond covalent radii for Ga and other 4p elements,^39^ a phenomenon known as d-orbital or scandide contraction.^40–42^ Indium, in contrast, exhibits significantly longer ionic and covalent radii.^39^ A key result of the present study is that it effectively quantifies the role of scandide contraction in modulating the electronic properties of (as opposed to bond distances in) several series of trielylenes.

## Results and Discussion

Five classes of neutral complexes have been examined in this study, based on β-diketiminate, bis­(imino)­carbazolate (pincer), hydrotrispyrazolylborate, cyclopentadienide, and monocoordinate aryl ligands. We have also examined a set of *N*,*N*′-diaryl-1,4-diazabutadiene dianion-coordinated triel­(I) anions (Chart 1). Vertical and adiabatic ionization potentials were calculated for each species; for several species, we also calculated electron affinities and singlet–triplet gaps. Two well-tested, scalar-relativistic DFT methods, OLYP^43,44^ -D3 ^45,46^ and B3LYP*,^47,48^ were used throughout. The results illustrate a rich variety of electronic effects, as a function of the metal, the ligand, and the interaction of the two, as described below.

**Chart 1:**
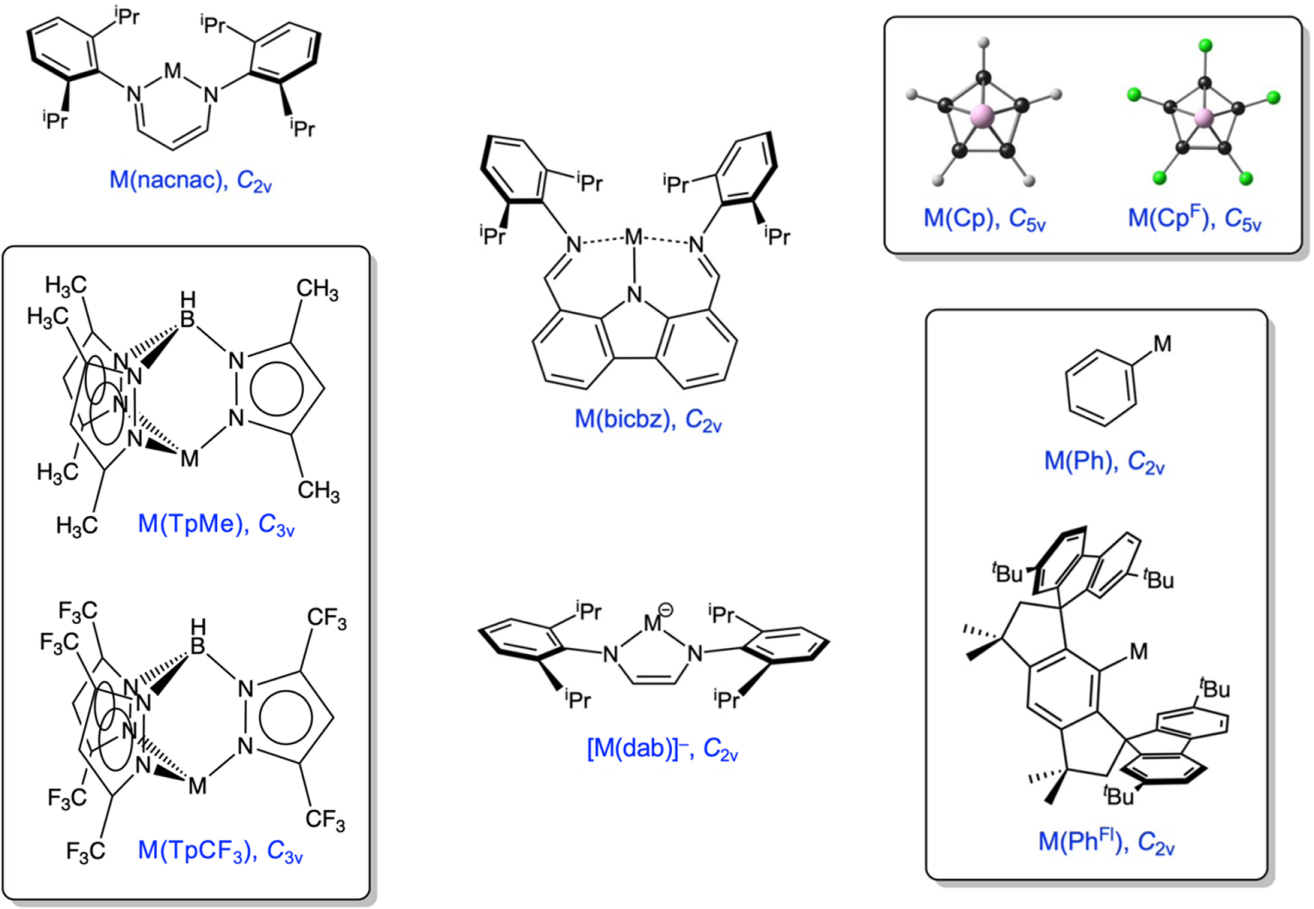
Six Classes of Trielylenes Examined in This Study[Fn c1fn1]

a (a) a β-diketiminate series, M­(nacnac) (*C*_2*v*_), (b) a bis­(imino)­carbazolate series, M­(bicbz) (*C*_2*v*_), (c) two hydrotrispyrazolylborate series, M­(TpMe) and M­(TpCF_3_) (*C*_3*v*_), (d) two cyclopentadienide series, M­(Cp) and M­(Cp^F^) (*C*_5*v*_), (e) a triyl or trielenyl anion series, [M­(dab)]^−^, where dab^2–^ is the 1,4-bis­(2′,6′-diisopropylphenyl)-1,4-diazabutadiene dianion, and (f) two aryltrielylenes series, M­(Ph) and M­(Ph^Fl^), All with M = Al, Ga and In. Hydrogen atoms have been generally omitted for clarity, except for M­(Cp).

### β-Diketiminate Complexes, M­(nacnac)

Sterically hindered β-diketiminate ligands have played a major role in the synthesis and isolation of monovalent aluminum and gallium complexes.^11,49^ We will refer to such ligands simply as nacnac in this study; Chart 1 depicts the exact structure used in our calculations. As alluded to above, the first Al­(nacnac) and Ga­(nacnac) complexes reported, respectively, by Roesky and Power and their co-workers in the year 2000;^7,8^ the subfield has also been recently reviewed.^11,15^ In our DFT survey of the field, the M­(nacnac) (M = Al, Ga, In) complexes yielded some of the most clear-cut results and accordingly are chosen as the paradigms in our discussion.

In general, the lowest ionization potential was found to correspond to ionization of the triel-based lone pair (Figures 1, 2 and 3). As shown in Table 1, both the adiabatic and vertical IPs (IP_1_ in Table 1) exhibit the following order: Ga > In ≫ Al. In other words, going down Group 13, the valence s-type lone pair is strongly stabilized from Al to Ga and slightly destabilized from Ga to In, consistent with a dramatic electronic manifestation of scandide contraction.

**Figure 1:**
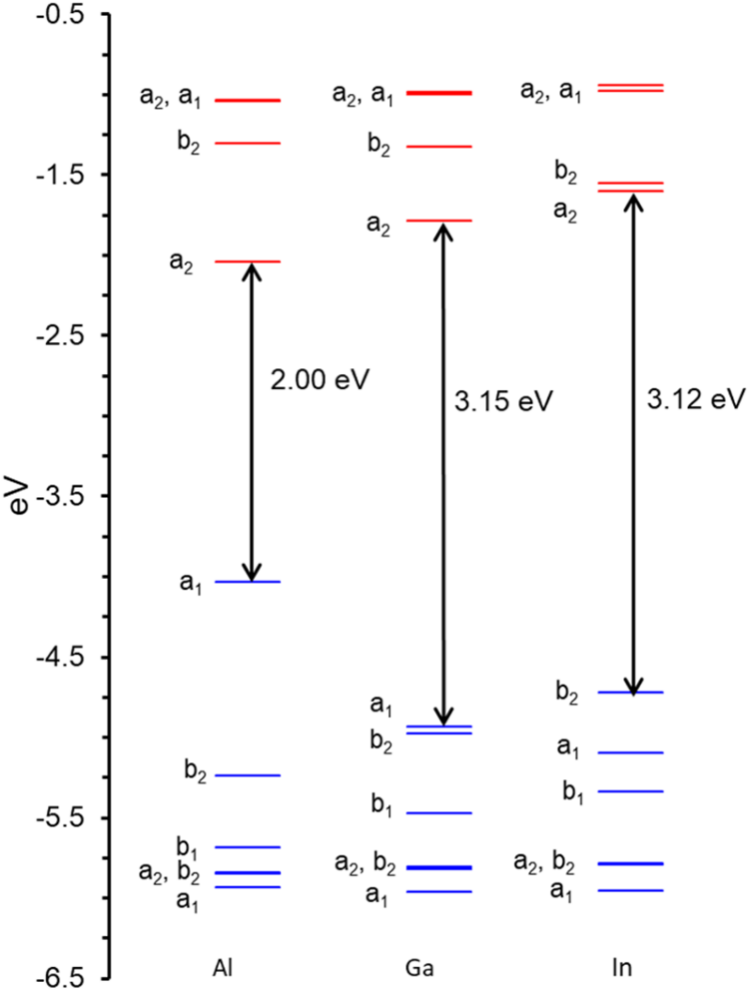
OLYP-D3/ZORA-STO-TZ2P frontier MO energy level diagrams for M­(nacnac), where M = Al, Ga, and In. The irreps refer to the *C*_2*v*_ point group.

**Figure 2:**
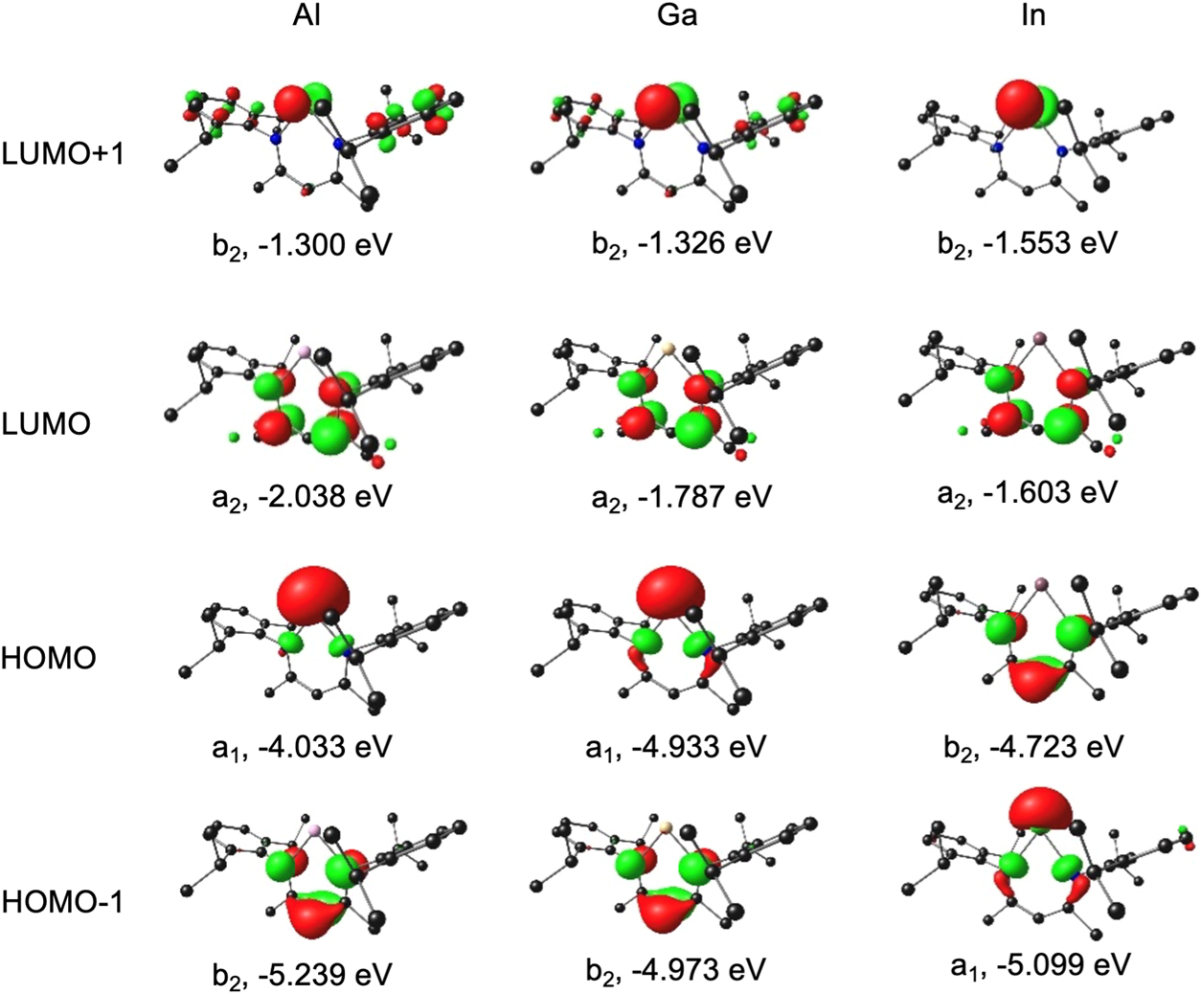
OLYP-D3/ZORA-STO-TZ2P frontier MOs of M­(nacnac) for M = Al, Ga, and In, along with *C*_2*v*_ irreps and orbital energies. Hydrogen atoms are omitted for clarity. Contour = 0.06 e/Å^3^.

**Figure 3:**
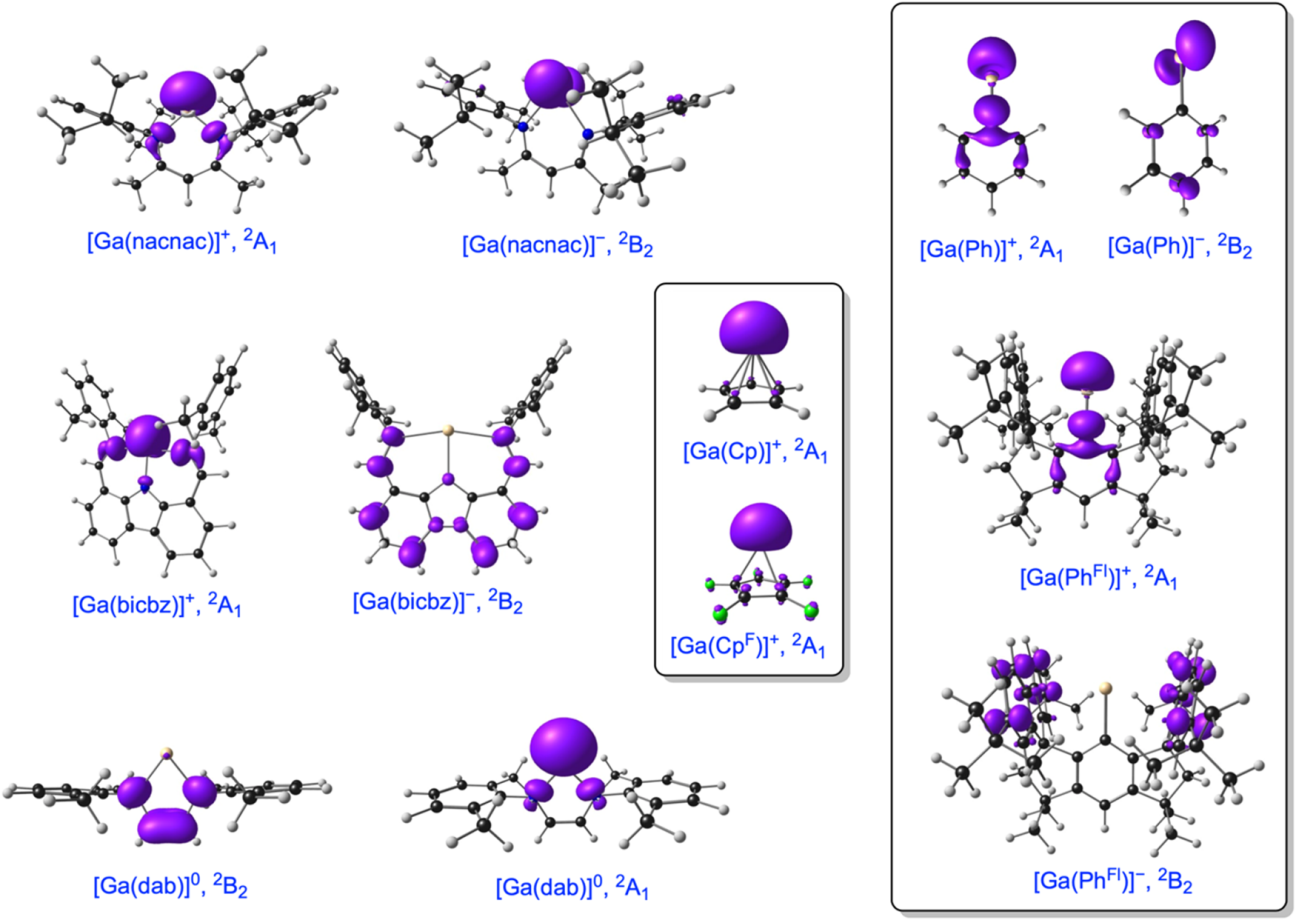
OLYP-D3/ZORA-STO-TZ2P spin density profiles plots for selected open-shell states of selected Ga complexes. The spin density profiles for the analogous Al and In species are, in general, visually quite similar. The irreps refer to the *C*_2v_ point group, except for the Cp and Cp^F^ complexes, where they refer to the *C*_5*v*_ point group. Contour = 0.004 e Å^–3^.

**Table 1:** Scalar-Relativistic OLYP-D3 and B3LYP* M–N Distances (*d*_M–N_), Ionization Potentials (IP), Electron Affinities (EA), and Singlet–Triplet Gaps (*E*_S–T_) for M­(nacnac) for M = Al, Ga, In^a^

			adiabatic energies (eV)				vertical energies (eV)			
metal	functional	*d*_M–N_ (Å)	IP_1_	IP_2_	EA	*E*_S–T_	IP_1_	IP_2_	EA_1_	*E*_S–T_
Al	OLYP-D3	2.051	5.45	6.88	0.20	1.54	6.06	6.99	–0.05	2.69
Ga	OLYP-D3	2.145	6.30	6.52	–0.04	2.48	6.72	6.77	–0.25	3.54
In	OLYP-D3	2.384	6.23	6.52	–0.17	2.66	6.55	6.72	–0.36	2.80
Al	B3LYP*	2.006	5.60	7.10	0.36	1.51	6.15	7.29	0.05	2.63
Ga	B3LYP*	2.124	6.53	6.80	0.11	2.49	7.04	7.00	–0.19	3.67
In	B3LYP*	2.339	6.57	6.77	0.00	2.68	6.77	7.06	–0.29	2.85

a For each species, IP1 and IP2 correspond to removal of an electron from the HOMO and HOMO – 1, respectively. The EA corresponds to addition of an electron to the LUMO, while the triplet energy corresponds to HOMO-to-LUMO excitation.

Interestingly, the second IPs, which corresponds to ionization of the nacnac π-system, exhibit a different trend relative to the first IPs, namely Al > Ga ≈ In (Table 1). This difference is most plausibly explained in terms of the aromaticity of the 6π-electron M-nacnac ring system. The smaller size of the Al atom leads to better π-overlap with the nacnac ligand and overall higher aromaticity of the chelate. The effect manifests itself in both a lower-energy HOMO – 1 and a higher-energy lowest unoccupied molecular orbital (LUMO) for the Al complex relative to its heavier congeners (Figures 1 and 2).

As shown in Table 1, the singlet–triplet gaps exhibit yet a different order: In ≳ Ga > Al. For all three complexes, the triplet states are derived by excitation of an electron from the valence s-type lone pair to a nacnac-based π-LUMO (Figure 2). While the Ga complex exhibits the lowest-energy HOMO ( reflecting scandide contraction), the LUMOs follow a different order, In > Ga > Al. The ordering of the triplet states thus reflects two different effects, with that of scandide contraction prevailing.

Consistent with our earlier findings, the OLYP-D3 and B3LYP* IPs are in fair agreement, differing by about 0.2 eV for the M­(nacnac) series. For some of the other classes of complexes examined in this study, the discrepancies between the two functionals are larger, on the order of 0.5 eV. Currently, we do not know which functional is better, given the paucity of experimental photoelectron spectroscopic data on low-valent p-block element compounds. For now, however, we are happy to “live with” the current level of accuracy; both functionals exhibit the same periodic trends and nearly identical differences in IPs among the Al, Ga, and In complexes in a given series.

Finally, as far as the M–N bond distances are concerned, the Ga–N bonds are about 0.1 Å longer than the Al–N bonds; the In–N bonds more significantly longer, by about 1/4-Å relative to the Ga–N bonds. These distances may be viewed as qualitatively consistent with scandide contraction (Table 1).

### Bis­(imino)­carbazolate Complexes, M­(bicbz)

A sterically hindered bis­(imino)­carbazolate (bicbz) pincer ligand provides an alternative to the much-used nacnac ligand for relatively low-valent metal complexes. Thus, Tan and co-workers recently reported the successful synthesis of Ga­(bicbz), but also noted their inability to generate the corresponding Al complex.^50^ In our calculations on M­(bicbz), we found the following order for IPs: In > Ga ≫ Al. However, a couple of special circumstances apply to these complexes, relative to their nacnac counterparts. The absolute values of the IPs are by far the lowest, for the four neutral trielylene series examined in this study (Table 2); the low IPs presumably reflect the strongly σ-donating nature of the anionic pincer ligand. A second interesting effect is that the Al complex exhibits a near-zero singlet–triplet gap. This, too, can be viewed as a consequence of the strongly σ-donating bicbz ligand: destabilization of the Al-based σ orbital leads to easy excitation into the ligand-based LUMO. (The nature of the metal-based HOMO and the ligand-based LUMO and the spin densities of the ionized states are visually rather similar for all three metals; see Figures 3 and 4). Together, the calculated results suggest that Al­(bicbz) may be unstable and inaccessible as a synthetic target, qualitatively consistent with Tan and co-workers’ findings.^50^

**Figure 4:**
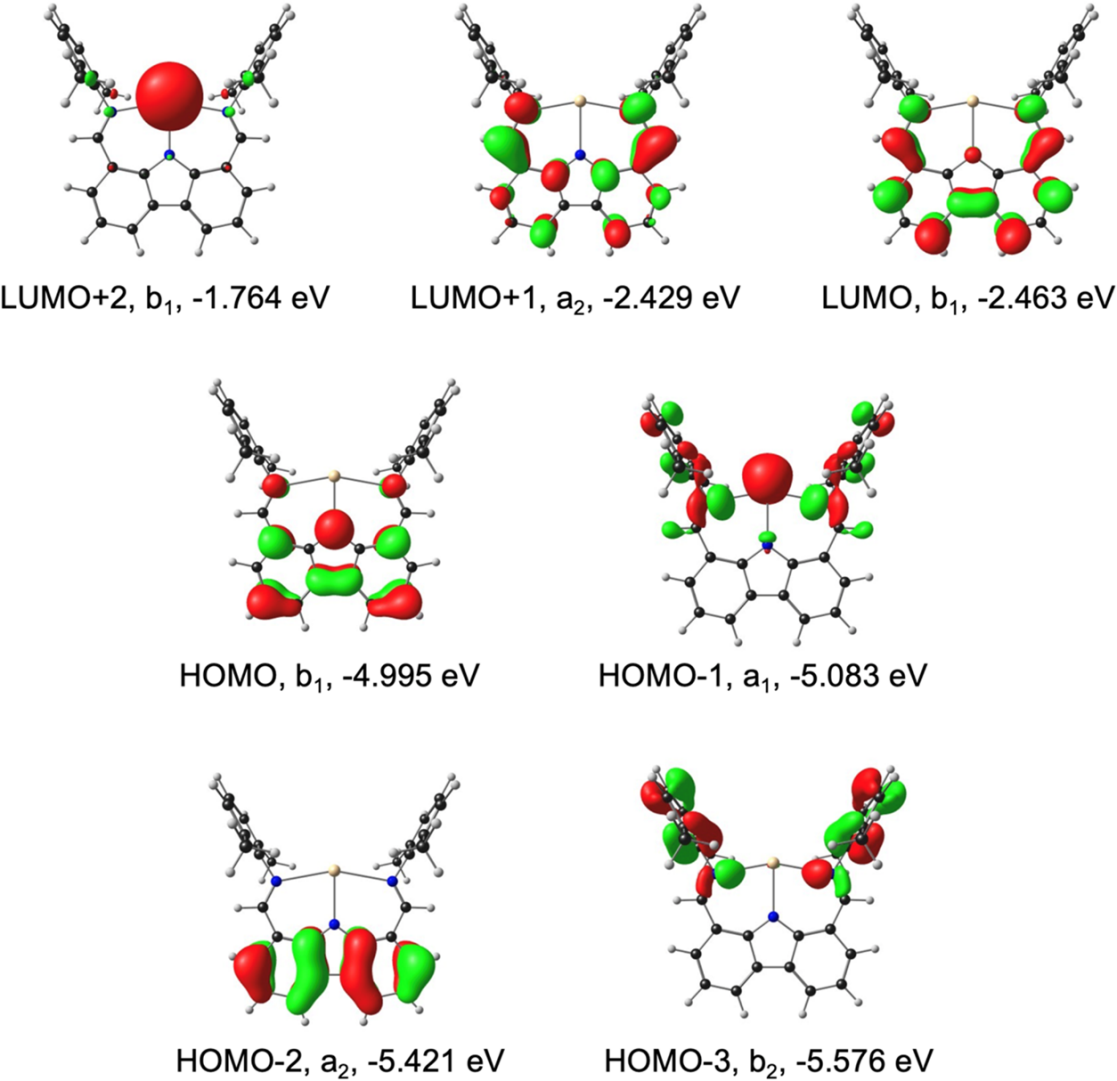
OLYP-D3/ZORA-STO-TZ2P frontier MOs of Ga­(bicbz), with *C*_2v_ irreps and orbital energies. Contour = 0.04 e/Å^3^.

**Table 2:** Scalar-Relativistic OLYP-D3 and B3LYP* M–N Distances (*d*_M–N_), Ionization Potentials (IP), Electron Affinities (EA), and Singlet–Triplet Gaps (*E*_S–T_) for the Bis­(imino)­carbazolate Series, M­(bicbz), with M = Al, Ga, and In

		*d*_M–N_ (Å)		adiabatic energies (eV)			vertical energies (eV)		
metal	functional	*N*_carbazolate_	*N*_imino_	IP	EA	*E*_S–T_	IP_1_	EA_1_	*E*_S–T_
Al	OLYP-D3	2.081	2.475	4.46	0.92	0.00	5.57	0.81	1.40
Ga	OLYP-D3	2.365	2.596	5.82	0.73	1.50	6.61	0.64	2.46
In	OLYP-D3	2.682	2.774	6.07	0.70	1.88	6.66	0.62	2.38
Al	B3LYP*	2.049	2.399	4.55	0.88	0.10	5.58	0.74	1.61
Ga	B3LYP*	2.174	2.499	5.84	0.77	1.57	6.60	0.66	2.67
In	B3LYP*	2.404	2.628	6.12	0.75	1.99	6.68	0.64	2.69

### Two Hydrotrispyrazolylborate Series, M­(TpMe) and M­(TpCF_3_)

Parkin and co-workers reported early examples of monovalent Ga, In, and Tl complexes with a pyramidal geometry based on hydrotrispyrazolylborate (Tp) ligands.^51–53^ Interestingly, they did not report an analogous Al complex. Although these authors used *t*-butyl-appended Tp ligands, we have modeled the complexes here with less sterically hindered methyl- and CF_3_-appended Tp ligands that lead to perfectly *C*_3v_-symmetric minimum-energy structures. For each complex studied, the HOMO corresponds to a triel-based s-type lone pair (Figure 5). Both OLYP and B3LYP* calculations indicated exceedingly low IPs for Al­(TpMe) (Table 3), echoing similar findings on Al­(bicbz) (see previous section); Al­(TpMe), accordingly, may be a very challenging, indeed potentially impossible, synthetic target. For Al­(TpCF_3_), however, the calculated adiabatic IP is over an eV higher than that of Al­(TpMe) and comparable to that of Ga­(TpMe), suggesting that Al­(TpCF_3_) may be a more tractable synthetic target.

**Figure 5:**
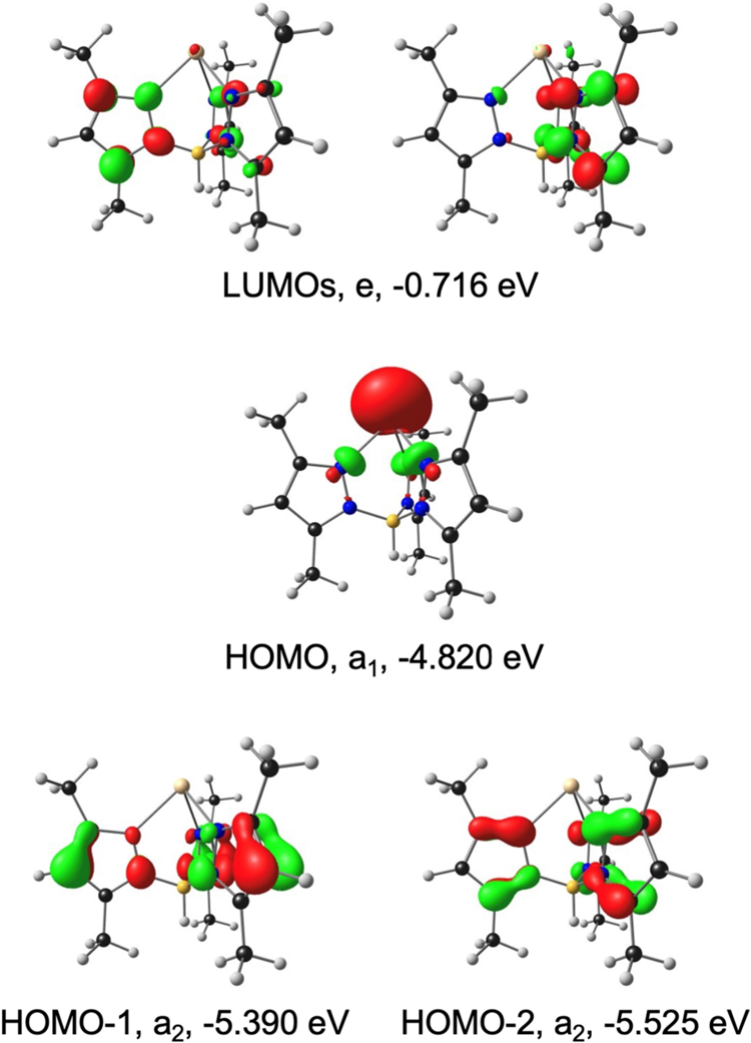
OLYP-D3/ZORA-STO-TZ2P frontier MOs of Ga­(TpMe), with *C*_3v_ irreps and orbital energies. Contour = 0.04 e/Å^3^.

**Table 3:** Scalar-Relativistic OLYP-D3 and B3LYP* M–N Distances (*d*_M–N_) and Adiabatic (IP_a_) and Vertical Ionization Potentials (IP_v_) for M­(TpMe) and M­(TpCF_3_), with M = Al, Ga, and In

		M(TpMe)			M(TpCF_3_)		
metal	functional	*d*_M–N_ (Å)	IP_a_	IP_v_	*d*_M–N_ (Å)	IP_a_	IP_v_
Al	OLYP-D3	2.110	4.56	5.35	2.359	5.77	7.39
Ga	OLYP-D3	2.324	5.75	7.11	2.547	7.44	9.14
In	OLYP-D3	2.537	6.16	7.15	2.789	7.73	8.81
Al	B3LYP*	2.094	4.73	5.47	2.295	5.96	7.39
Ga	B3LYP*	2.246	5.94	6.99	2.433	7.57	9.14
In	B3LYP*	2.442	6.35	7.13	2.630	7.83	9.05

### Diazabutadiene Chelate Anions, [M­(dab)]^−^

In recent years, so-called “aluminyl” and “gallyl” anions have emerged as new motifs in the chemistry of low-valent Group 13 compounds. In this study, we have examined a series of sterically hindered, anionic 1,4-diazabutadiene chelates [M­(dab)]^−^.^54–56^ From Chart 1, note that the ligand is dianionic and that the M­(I) center carries a formal charge of −1. The valence of the metal center, accordingly, is: number of bonds + formal charge = 2 + (−1) = 1. The lowest IPs of the dab-based anions vary in the order Al > Ga > In and correspond to ionization of a ligand-based π-HOMO (Table 4 and Figure 6). The order can be explained in terms of the aromaticity of the MC_2_N_2_ ring, which may be viewed as isoelectronic to the *N*-heterocyclic carbene imidazol-2-ylidene. As in the M­(nacnac) series, the Al complex is most aromatic, with the lowest-energy π-HOMO, which explains its relatively high IP. The second IPs correspond to ionization of the metal-based lone pair and follow the order In > Ga ≫ Al, consistent with scandide contraction.

**Figure 6:**
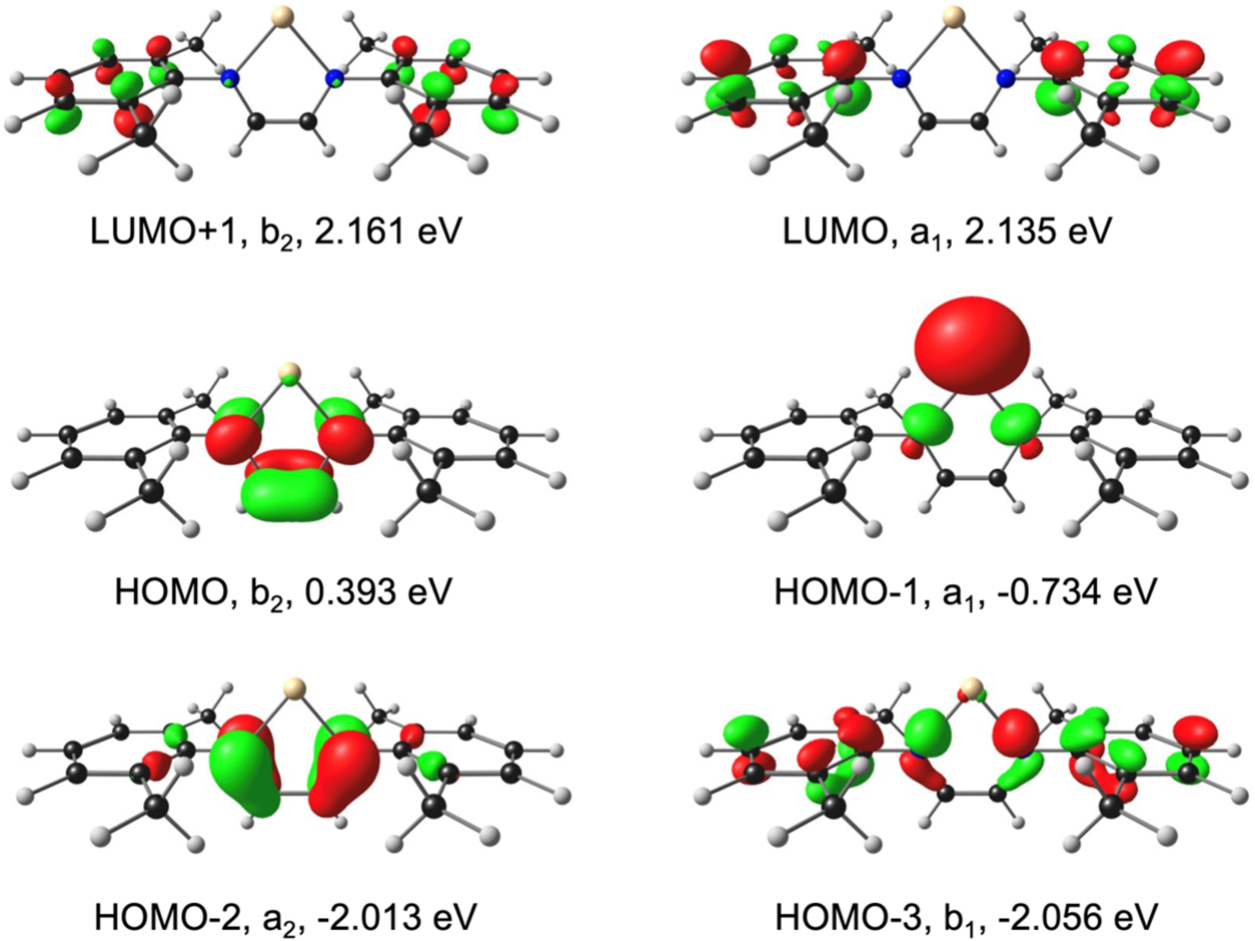
OLYP-D3/ZORA-STO-TZ2P frontier MOs of [Ga­(dab)]^−^, with *C*_2v_ irreps and orbital energies. Contour = 0.04 e/Å^3^.

**Table 4:** Scalar-Relativistic OLYP-D3 and B3LYP* M–N Distances (*d*_M–N_) and Ionization Potentials for [M­(dab)]^−^ for M = Al, Ga, and In^a^

			adiabatic IPs (eV) for neutral final states		vertical IPs (eV) for neutral final states	
metal	functional	*d*_M–N_ (Å)	^2^B_2_^0^	^2^A_1_^0^	^2^B_2_^0^	^2^A_1_^0^
Al	OLYP-D3	1.984	1.65	1.79	1.95	2.22
Ga	OLYP-D3	2.064	1.34	2.38	1.64	2.73
In	OLYP-D3	2.288	1.16	2.69	1.40	2.91
Al	B3LYP*	1.937	1.82	1.93	2.14	2.26
Ga	B3LYP*	2.042	1.51	2.58	1.82	2.94
In	B3LYP*	2.256	1.34	2.91	1.62	3.14

a For the IPs, two different final state symmetries were considered.

### Cyclopentadienyl Half-Sandwich Complexes, M­(Cp) and M­(Cp^F^)

The first cyclopentadienyltriels, In­(Cp) and Tl­(Cp), were synthesized in the middle of the last century and, at the time, were among the first organotriels to be reported.^57–59^ In 1991, Dohmeier reported Al­(Cp*), the first example of a room-temperature-stable Al­(I) compound.^6^ In its isolated form, the compound was found to exist as a tetramer. Gas-phase electron diffraction experiments by Haaland and co-workers, however, provided full structural details of the monomeric form.^60^ The 1990s also saw the synthesis and structural characterization of Al­(Cp),^61^ Ga­(Cp*),^62,63^ and Ga­(Cp).^64^ Herein, we have studied two series of *C*_5v_-symmetric complexes M­(Cp) and M­(Cp^F^) (M = Al, Ga, In).

The IPs of the cyclopentadienide series (Table 5) follow the same order as those of the nacnac series, i.e., Ga > In ≫ Al, consistent with a significant electronic effect attributable to scandide contraction. The ionization involves removal of an electron from the a_1_ irreps, i.e., from the valence s-type lone pair on the metal, as evident from the spin density of the cationic states (Figure 3). The M–C bond distances, on the other hand, increase in the order Al < Ga < In and do not reflect a strong effect attributable to scandide contraction.

**Table 5:** Scalar-Relativistic OLYP-D3 and B3LYP* M–C Distances (*d*_M–C_) and Adiabatic and Vertical Ionization Potentials for M­(Cp) and M­(Cp^F^) for M = Al, Ga, and In

compound	functional	*d*_M–C_ (Å)	adiabatic IP (eV)	vertical IP (eV)
Al(Cp)	OLYP	2.355	7.29	7.74
Ga(Cp)	OLYP	2.534	8.25	9.06
In(Cp)	OLYP	2.740	7.97	8.58
Al(Cp)	B3LYP*	2.388	7.60	8.15
Ga(Cp)	B3LYP*	2.471	8.63	9.24
In(Cp)	B3LYP*	2.663	8.31	8.76
Al(Cp^F^)	OLYP	2.402	8.42	8.80
Ga(Cp^F^)	OLYP	2.656	9.27	9.95
In(Cp^F^)	OLYP	2.886	8.94	9.50
Al(Cp^F^)	B3LYP*	2.441	8.86	9.37
Ga(Cp^F^)	B3LYP*	2.536	9.81	10.34
In(Cp^F^)	B3LYP*	2.740	9.41	9.84

### Aryltrielylenes, M­(Ph) and M­(Ph^Fl*^)

Among the complexes examined in this study, aryltrielylenes M­(Ar) are unique in that the valence, oxidation state, and coordination number of the metal each equals one. Power and co-workers were the first to report monocoordinate In­(Ar) and Tl­(Ar) complexes in 1998.^65,66^ Subsequently, using bulky terphenyl-based ligands, the same group also successfully synthesized Ga­(Ar)^67^ and Al­(Ar)^68^ complexes. Very recently, an alternative fluorenylidene-flanked architecture was reported for a Ga­(Ar) complex.^69^ We have modeled these complexes here using both unadorned M­(Ph) complexes and M­(Ph^Fl*^), where Ph^Fl*^ refers to the fluorenylidene-flanked aryl ligand used in the recent study.

The results of our calculations on aryltrielylenes proved somewhat of a surprise relative to the rest of our findings (Table 6). Although the IPs for each series follow the order Ga > In > Al, qualitatively consistent with scandide contraction, the differences among the three elements are found to be remarkably muted. For the M­(Ph) series, the IPs all fall within a narrow band of 0.2 eV, while for M­(Ph^Fl*^), they fall within a marginally wider range of 0.4 eV. The reason for the muted differences among the three triels appears to lie in the nature of the molecules’ HOMOs. These orbitals cannot be described as metal-centered lone pairs but are better described as occupied M–C σ-antibonding MOs with varying admixtures of phenyl character. Aside from that and unsurprisingly, the IPs of the M­(Ph^Fl*^) series are distinctly lower than those of unadorned M­(Ph), reflecting the electron-donating effect of the Ph^Fl*^ framework.

**Table 6:** Scalar-Relativistic OLYP-D3 and B3LYP* Ionization Potentials (IP), Electron Affinities (EA), and Singlet–Triplet (S–T) Gaps (*E*_S–T_) for Aryltrielylenes M­(Ar) for M = Al, Ga, and In

			adiabatic energies (eV)			vertical energies (eV)		
molecule	functional	*d*_M–C_ (Å)	IP	EA	*E*_S–T_	IP_1_	EA_1_	*E*_S–T_
Al(Ph)	OLYP-D3	2.018	7.20	0.19	1.88	7.29	0.17	1.91
Ga(Ph)	OLYP-D3	2.076	7.27	0.05	2.15	7.41	0.04	2.21
In(Ph)	OLYP-D3	2.281	6.93	0.08	2.09	7.08	0.05	2.14
Al(Ph)	B3LYP*	2.005	7.41	0.22	2.09	7.50	0.20	2.11
Ga(Ph)	B3LYP*	2.036	7.66	0.14	2.37	7.66	0.14	2.37
In(Ph)	B3LYP*	2.270	7.18	0.28	2.19	7.35	0.26	2.25
Al(Ph^Fl^)	OLYP-D3	2.080	5.36	0.32	1.64	5.76	0.25	1.72
Ga(Ph^Fl^)	OLYP-D3	2.147	5.77	0.31	2.05	6.31	0.25	2.18
In(Ph^Fl^)	OLYP-D3	2.407	5.70	0.31	2.13	6.30	0.25	2.26
Al(Ph^Fl^)	B3LYP*	2.061	5.58	0.19	1.86	6.18	0.13	1.95
Ga(Ph^Fl^)	B3LYP*	2.120	6.02	0.17	2.21	6.42	0.12	2.32
In(Ph^Fl^)	B3LYP*	2.343	5.89	0.21	2.20	6.21	0.19	2.31

## Conclusions

A DFT study of 6 classes of monovalent Group 13 species or trielylenes (involving Al, Ga, and In) has helped us delineate periodic trends in their electronic properties and underscored the significant impact of scandide contraction on the Ga species. In general, compared with their aluminum counterparts, the metal-based lone pairs of monovalent Ga species are dramatically stabilized; the corresponding IPs are about three-quarters of an eV to well over an eV higher for the gallium species. The corresponding IPs for the In species are generally similar to those of their Ga counterpart or slightly lower. The monovalent metal-aryls are an exception to this generalization. The metal centers in these molecules do not harbor a true s-type lone pair; instead, the HOMO is an occupied M–C antibonding (σ*) orbital. Understandably, scandide contraction has a relatively muted effect for ionization of such an orbital relative to a metal-based lone pair.

## Computational Methods

Scalar-relativistic ZORA^70^ DFT calculations were carried out with the OLYP^43,44^ -D3 ^45,46^ and B3LYP* ^47,48^ methods, all-electron ZORA STO-TZ2P basis sets, and carefully tested, tight criteria for SCF and geometry cycles, all as implemented in the ADF 2019 program system.^71^ Point group symmetry was employed so as to enable electron removal from (or electron addition to) specific irreps.

## Supplementary Material



## Data Availability

All data generated or analyzed in this study are included in this published article and its Supporting Information.

## Notes

Optimized DFT coordinates (PDF)
