# Supplementary material for: Electronic Manifestations of Scandide Contraction: Theoretical Photoelectron Spectroscopy of Monovalent Group 13 Compounds
Source: Inorg Chem. 2025 Jul 12;64(29):15098–106. doi: 10.1021/acs.inorgchem.5c02000 (PMC12308808; doi:10.1021/acs.inorgchem.5c02000)
Supplement: Supplementary file 1 [file ic5c02000_si_001.pdf]

Supporting information

Electronic Manifestations of Scandide Contraction: Theoretical Photoelectron Spectroscopy of Monovalent Group 13 Compounds

Jeanet Conradie[a,b], Kristian Torstensen,[a] Pekka Pyykkö[c] and Abhik Ghosh*,[a]

[a] Department of Chemistry, UiT – The Arctic University of Norway, N-9037 Tromsø, Norway; Email: abhik.ghosh@uit.no (AG)
[b] Department of Chemistry, University of the Free State, P.O. Box 339, Bloemfontein 9300, Republic of South Africa.
[c] Department of Chemistry, Faculty of Science, University of Helsinki, POB 55, 00014 Helsinki, Finland.'

Optimized Cartesian coordinates (Å)

All compounds were optimized the ZORA Hamiltonian, the OLYP functional including Grimme's D3 dispersion correction, and all-electron ZORA/TZ2P basis sets.

Table of Contents

Table of Contents 1
Mnacnac 3
1. Al(nacnac), C_{2v}, q = 0, S = 0 3
2. Ga(nacnac), C_{2v}, q = 0, S = 0 4
3. In(nacnac), C_{2v}, q = 0, S = 0 6
Cyclopentadienide series 7
4. AlCp, C_{5v}, q = 0, S = 0 7
5. GaCp, C_{5v}, q = 0, S = 0 8
6. InCp, C_{5v}, q = 0, S = 0 8
7. Al(Cp^F), C_{5v}, q = 0, S = 0 8

8. Ga(Cp^F), C_{5v}, q = 0, S = 0 8
9. In(Cp^F), C_{5v}, q = 0, S = 0 9
Bis(imino)carbazolate 9
10. Al(bicbz), C_{2v}, q = 0, S = 0 9
11. Ga(bicbz), C_{2v}, q = 0, S = 0 10
12. In(bicbz), C_{2v}, q = 0, S = 0 12
[M(dab)]$^-$ 13
13. [Al(dab)]$^-$, C_{2v}, q = -1, S = 0 13
14. [Ga(dab)]$^-$, C_{2v}, q = -1, S = 0 14
15. [In(dab)]$^-$, C_{2v}, q = -1, S = 0 15
Aryltrielylenes 16
16. Al(Ph^{Fl}), C_{2v}, q = 0, S = 0 16
17. Ga(Ph^{Fl}), C_{2v}, q = 0, S = 0 18
18. In(Ph^{Fl}), C_{2v}, q = 0, S = 0 21
Hydrotrispyrazolylborates 23
19. Al(TpMe), C_{3v}, q = 0, S = 0 23
20. Ga(TpMe), C_{3v}, q = 0, S = 0 24
21. In(TpMe), C_{3v}, q = 0, S = 0 25
22. Al(TpCF$_3$), C_{3v}, q = 0, S = 0 26
23. Ga(TpCF$_3$), C_{3v}, q = 0, S = 0 27
24. In(TpCF$_3$), C_{3v}, q = 0, S = 0 28

Mnacnac

1. Al(nacnac), C_{2v}, q = 0, *S* = 0

Al	0.000000000	0.000000000	-1.067776000
C	0.000000000	0.000000000	2.367562000
C	1.255025000	0.000000000	1.746268000
C	2.294152000	3.273556000	-1.328304000
C	2.294152000	-3.273556000	-1.328304000
C	2.474517000	0.000000000	2.635914000
C	2.730660000	2.553775000	-0.042815000
C	2.730660000	-2.553775000	-0.042815000
C	2.764221000	0.000000000	-0.089627000
C	3.398604000	1.228214000	-0.363472000
C	3.398604000	-1.228214000	-0.363472000
C	3.625733000	3.462666000	0.813209000
C	3.625733000	-3.462666000	0.813209000
C	4.665921000	1.204460000	-0.952578000
C	4.665921000	-1.204460000	-0.952578000
C	5.296361000	0.000000000	-1.253009000
C	-1.255025000	0.000000000	1.746268000
C	-2.294152000	3.273556000	-1.328304000
C	-2.294152000	-3.273556000	-1.328304000
C	-2.474517000	0.000000000	2.635914000
C	-2.730660000	2.553775000	-0.042815000
C	-2.730660000	-2.553775000	-0.042815000
C	-2.764221000	0.000000000	-0.089627000
C	-3.398604000	1.228214000	-0.363472000
C	-3.398604000	-1.228214000	-0.363472000
C	-3.625733000	3.462666000	0.813209000
C	-3.625733000	-3.462666000	0.813209000
C	-4.665921000	1.204460000	-0.952578000
C	-4.665921000	-1.204460000	-0.952578000
C	-5.296361000	0.000000000	-1.253009000
H	0.000000000	0.000000000	3.450802000
H	1.623245000	2.640660000	-1.917390000
H	1.623245000	-2.640660000	-1.917390000
H	1.774295000	4.207125000	-1.082973000
H	1.774295000	-4.207125000	-1.082973000
H	1.829726000	2.339402000	0.536931000
H	1.829726000	-2.339402000	0.536931000
H	2.194999000	0.000000000	3.691718000
H	3.075273000	4.366343000	1.098781000
H	3.075273000	-4.366343000	1.098781000
H	3.093156000	0.876917000	2.423681000
H	3.093156000	-0.876917000	2.423681000
H	3.167456000	3.514057000	-1.944692000
H	3.167456000	-3.514057000	-1.944692000

H	3.954514000	2.953410000	1.724927000
H	3.954514000	-2.953410000	1.724927000
H	4.517402000	3.773522000	0.259341000
H	4.517402000	-3.773522000	0.259341000
H	5.163847000	2.142533000	-1.180096000
H	5.163847000	-2.142533000	-1.180096000
H	6.281178000	0.000000000	-1.715187000
H	-1.623245000	2.640660000	-1.917390000
H	-1.623245000	-2.640660000	-1.917390000
H	-1.774295000	4.207125000	-1.082973000
H	-1.774295000	-4.207125000	-1.082973000
H	-1.829726000	2.339402000	0.536931000
H	-1.829726000	-2.339402000	0.536931000
H	-2.194999000	0.000000000	3.691718000
H	-3.075273000	4.366343000	1.098781000
H	-3.075273000	-4.366343000	1.098781000
H	-3.093156000	0.876917000	2.423681000
H	-3.093156000	-0.876917000	2.423681000
H	-3.167456000	3.514057000	-1.944692000
H	-3.167456000	-3.514057000	-1.944692000
H	-3.954514000	2.953410000	1.724927000
H	-3.954514000	-2.953410000	1.724927000
H	-4.517402000	3.773522000	0.259341000
H	-4.517402000	-3.773522000	0.259341000
H	-5.163847000	2.142533000	-1.180096000
H	-5.163847000	-2.142533000	-1.180096000
H	-6.281178000	0.000000000	-1.715187000
N	1.416028000	0.000000000	0.416300000
N	-1.416028000	0.000000000	0.416300000

2. Ga(nacnac), C_{2v}, q = 0, S = 0

Ga	0.000000000	0.000000000	-1.012573000
C	0.000000000	0.000000000	2.495217000
C	1.263949000	0.000000000	1.885113000
C	2.251620000	3.253099000	-1.215688000
C	2.251620000	-3.253099000	-1.215688000
C	2.475549000	0.000000000	2.789778000
C	2.730116000	2.549019000	0.063985000
C	2.730116000	-2.549019000	0.063985000
C	2.780226000	0.000000000	0.042959000
C	3.402773000	1.227781000	-0.263020000
C	3.402773000	-1.227781000	-0.263020000
C	3.637085000	3.475712000	0.887042000
C	3.637085000	-3.475712000	0.887042000
C	4.647810000	1.204847000	-0.897536000
C	4.647810000	-1.204847000	-0.897536000
C	5.269655000	0.000000000	-1.217068000
C	-1.263949000	0.000000000	1.885113000
C	-2.251620000	3.253099000	-1.215688000

C	-2.251620000	-3.253099000	-1.215688000
C	-2.475549000	0.000000000	2.789778000
C	-2.730116000	2.549019000	0.063985000
C	-2.730116000	-2.549019000	0.063985000
C	-2.780226000	0.000000000	0.042959000
C	-3.402773000	1.227781000	-0.263020000
C	-3.402773000	-1.227781000	-0.263020000
C	-3.637085000	3.475712000	0.887042000
C	-3.637085000	-3.475712000	0.887042000
C	-4.647810000	1.204847000	-0.897536000
C	-4.647810000	-1.204847000	-0.897536000
C	-5.269655000	0.000000000	-1.217068000
H	0.000000000	0.000000000	3.578589000
H	1.577014000	2.608647000	-1.787177000
H	1.577014000	-2.608647000	-1.787177000
H	1.723280000	4.180628000	-0.965704000
H	1.723280000	-4.180628000	-0.965704000
H	1.847304000	2.328557000	0.668545000
H	1.847304000	-2.328557000	0.668545000
H	2.188187000	0.000000000	3.843325000
H	3.086290000	4.377813000	1.176968000
H	3.086290000	-4.377813000	1.176968000
H	3.095643000	0.877643000	2.582445000
H	3.095643000	-0.877643000	2.582445000
H	3.105840000	3.501536000	-1.855049000
H	3.105840000	-3.501536000	-1.855049000
H	3.991724000	2.977726000	1.795198000
H	3.991724000	-2.977726000	1.795198000
H	4.512420000	3.787219000	0.307982000
H	4.512420000	-3.787219000	0.307982000
H	5.135218000	2.143095000	-1.146972000
H	5.135218000	-2.143095000	-1.146972000
H	6.237671000	0.000000000	-1.713457000
H	-1.577014000	2.608647000	-1.787177000
H	-1.577014000	-2.608647000	-1.787177000
H	-1.723280000	4.180628000	-0.965704000
H	-1.723280000	-4.180628000	-0.965704000
H	-1.847304000	2.328557000	0.668545000
H	-1.847304000	-2.328557000	0.668545000
H	-2.188187000	0.000000000	3.843325000
H	-3.086290000	4.377813000	1.176968000
H	-3.086290000	-4.377813000	1.176968000
H	-3.095643000	0.877643000	2.582445000
H	-3.095643000	-0.877643000	2.582445000
H	-3.105840000	3.501536000	-1.855049000
H	-3.105840000	-3.501536000	-1.855049000
H	-3.991724000	2.977726000	1.795198000
H	-3.991724000	-2.977726000	1.795198000
H	-4.512420000	3.787219000	0.307982000
H	-4.512420000	-3.787219000	0.307982000
H	-5.135218000	2.143095000	-1.146972000

H	-5.135218000	-2.143095000	-1.146972000
H	-6.237671000	0.000000000	-1.713457000
N	1.451945000	0.000000000	0.566005000
N	-1.451945000	0.000000000	0.566005000

3. In(nacnac), C_{2v}, q = 0, *S* = 0

In	0.000000000	0.000000000	-1.205612000
C	0.000000000	0.000000000	2.520658000
C	1.278762000	0.000000000	1.935639000
C	2.457393000	0.000000000	2.888631000
C	2.473222000	3.283377000	-1.106255000
C	2.473222000	-3.283377000	-1.106255000
C	2.834909000	2.551336000	0.196184000
C	2.834909000	-2.551336000	0.196184000
C	2.874144000	0.000000000	0.174395000
C	3.520633000	1.227098000	-0.092075000
C	3.520633000	-1.227098000	-0.092075000
C	3.678339000	3.459222000	1.104774000
C	3.678339000	-3.459222000	1.104774000
C	4.805433000	1.203946000	-0.641639000
C	4.805433000	-1.203946000	-0.641639000
C	5.448858000	0.000000000	-0.918643000
C	-1.278762000	0.000000000	1.935639000
C	-2.457393000	0.000000000	2.888631000
C	-2.473222000	3.283377000	-1.106255000
C	-2.473222000	-3.283377000	-1.106255000
C	-2.834909000	2.551336000	0.196184000
C	-2.834909000	-2.551336000	0.196184000
C	-2.874144000	0.000000000	0.174395000
C	-3.520633000	1.227098000	-0.092075000
C	-3.520633000	-1.227098000	-0.092075000
C	-3.678339000	3.459222000	1.104774000
C	-3.678339000	-3.459222000	1.104774000
C	-4.805433000	1.203946000	-0.641639000
C	-4.805433000	-1.203946000	-0.641639000
C	-5.448858000	0.000000000	-0.918643000
H	0.000000000	0.000000000	3.604302000
H	1.847577000	2.657101000	-1.749830000
H	1.847577000	-2.657101000	-1.749830000
H	1.903153000	2.330220000	0.722388000
H	1.903153000	-2.330220000	0.722388000
H	1.930595000	4.210239000	-0.885109000
H	1.930595000	-4.210239000	-0.885109000
H	2.132245000	0.000000000	3.931050000
H	3.085309000	0.876984000	2.705501000
H	3.085309000	-0.876984000	2.705501000
H	3.114503000	4.364585000	1.357885000
H	3.114503000	-4.364585000	1.357885000
H	3.379834000	3.538772000	-1.665884000

H	3.379834000	-3.538772000	-1.665884000
H	3.949638000	2.948206000	2.033784000
H	3.949638000	-2.948206000	2.033784000
H	4.603117000	3.766508000	0.605267000
H	4.603117000	-3.766508000	0.605267000
H	5.309604000	2.142672000	-0.854350000
H	5.309604000	-2.142672000	-0.854350000
H	6.448855000	0.000000000	-1.346664000
H	-1.847577000	2.657101000	-1.749830000
H	-1.847577000	-2.657101000	-1.749830000
H	-1.903153000	2.330220000	0.722388000
H	-1.903153000	-2.330220000	0.722388000
H	-1.930595000	4.210239000	-0.885109000
H	-1.930595000	-4.210239000	-0.885109000
H	-2.132245000	0.000000000	3.931050000
H	-3.085309000	0.876984000	2.705501000
H	-3.085309000	-0.876984000	2.705501000
H	-3.114503000	4.364585000	1.357885000
H	-3.114503000	-4.364585000	1.357885000
H	-3.379834000	3.538772000	-1.665884000
H	-3.379834000	-3.538772000	-1.665884000
H	-3.949638000	2.948206000	2.033784000
H	-3.949638000	-2.948206000	2.033784000
H	-4.603117000	3.766508000	0.605267000
H	-4.603117000	-3.766508000	0.605267000
H	-5.309604000	2.142672000	-0.854350000
H	-5.309604000	-2.142672000	-0.854350000
H	-6.448855000	0.000000000	-1.346664000
N	1.524233000	0.000000000	0.627501000
N	-1.524233000	0.000000000	0.627501000

Cyclopentadienide series

4. AlCp, C_{5v}, q = 0, S = 0

Al	0.000000000	0.000000000	1.581785000
C	-0.373457000	1.149382000	-0.439386000
C	-1.208532000	0.000000000	-0.439386000
C	0.977723000	0.710357000	-0.439386000
C	-0.373457000	-1.149382000	-0.439386000
C	0.977723000	-0.710357000	-0.439386000
H	1.853330000	-1.346523000	-0.415358000
H	1.853330000	1.346523000	-0.415358000
H	-0.707909000	2.178720000	-0.415358000
H	-2.290841000	0.000000000	-0.415358000
H	-0.707909000	-2.178720000	-0.415358000

5. GaCp, C_{5v}, q = 0, S = 0

Ga	0.000000000	0.000000000	1.785894000
C	-0.373642000	1.149951000	-0.440728000
C	-1.209130000	0.000000000	-0.440728000
C	0.978207000	0.710709000	-0.440728000
C	-0.373642000	-1.149951000	-0.440728000
C	0.978207000	-0.710709000	-0.440728000
H	1.854259000	-1.347198000	-0.454838000
H	1.854259000	1.347198000	-0.454838000
H	-0.708264000	2.179813000	-0.454838000
H	-2.291991000	0.000000000	-0.454838000
H	-0.708264000	-2.179813000	-0.454838000

6. InCp, C_{5v}, q = 0, S = 0

In	0.000000000	0.000000000	2.004241000
C	-0.374066000	1.151258000	-0.454022000
C	-1.210504000	0.000000000	-0.454022000
C	0.979318000	0.711516000	-0.454022000
C	-0.374066000	-1.151258000	-0.454022000
C	0.979318000	-0.711516000	-0.454022000
H	1.855332000	-1.347978000	-0.485214000
H	1.855332000	1.347978000	-0.485214000
H	-0.708674000	2.181074000	-0.485214000
H	-2.293317000	0.000000000	-0.485214000
H	-0.708674000	-2.181074000	-0.485214000

7. Al(Cp^F), C_{5v}, q = 0, S = 0

Al	0.000000000	0.000000000	1.656556000
C	-0.373341000	1.149026000	-0.419715000
C	-1.208158000	0.000000000	-0.419715000
C	0.977420000	0.710137000	-0.419715000
C	-0.373341000	-1.149026000	-0.419715000
C	0.977420000	-0.710137000	-0.419715000
F	2.056856000	-1.494393000	-0.449983000
F	2.056856000	1.494393000	-0.449983000
F	-0.785649000	2.417979000	-0.449983000
F	-2.542413000	0.000000000	-0.449983000
F	-0.785649000	-2.417979000	-0.449983000

8. Ga(Cp^F), C_{5v}, q = 0, S = 0

Ga	0.000000000	0.000000000	1.943270000
C	-0.374439000	1.152405000	-0.420272000
C	-1.211710000	0.000000000	-0.420272000
C	0.980294000	0.712225000	-0.420272000

C	-0.374439000	-1.152405000	-0.420272000
C	0.980294000	-0.712225000	-0.420272000
F	2.061157000	-1.497518000	-0.506769000
F	2.061157000	1.497518000	-0.506769000
F	-0.787292000	2.423035000	-0.506769000
F	-2.547730000	0.000000000	-0.506769000
F	-0.787292000	-2.423035000	-0.506769000

9. In(Cp^F), C_{5v}, q = 0, S = 0

In	0.000000000	0.000000000	2.181371000
C	-0.374285000	1.151930000	-0.437974000
C	-1.211210000	0.000000000	-0.437974000
C	0.979890000	0.711932000	-0.437974000
C	-0.374285000	-1.151930000	-0.437974000
C	0.979890000	-0.711932000	-0.437974000
F	2.061944000	-1.498090000	-0.536687000
F	2.061944000	1.498090000	-0.536687000
F	-0.787593000	2.423961000	-0.536687000
F	-2.548703000	0.000000000	-0.536687000
F	-0.787593000	-2.423961000	-0.536687000

Bis(imino)carbazolate

10. Al(bicbz), C_{2v}, q = 0, S = 0

Al	0.000000000	0.000000000	0.568740000
C	0.000000000	0.715988000	-3.728419000
C	0.000000000	1.115777000	-2.357751000
C	0.000000000	1.632901000	-4.777465000
C	0.000000000	2.509773000	-2.070183000
C	0.000000000	2.995492000	-4.487046000
C	0.000000000	3.116765000	-0.758432000
C	0.000000000	3.155906000	1.582150000
C	0.000000000	3.409853000	-3.157325000
C	0.000000000	4.371735000	4.082784000
C	0.000000000	-0.715988000	-3.728419000
C	0.000000000	-1.115777000	-2.357751000
C	0.000000000	-1.632901000	-4.777465000
C	0.000000000	-2.509773000	-2.070183000
C	0.000000000	-2.995492000	-4.487046000
C	0.000000000	-3.116765000	-0.758432000
C	0.000000000	-3.155906000	1.582150000
C	0.000000000	-3.409853000	-3.157325000
C	0.000000000	-4.371735000	4.082784000
C	1.206389000	4.060251000	3.457321000
C	1.206389000	-4.060251000	3.457321000
C	1.227710000	3.443146000	2.203625000
C	1.227710000	-3.443146000	2.203625000

C	2.521320000	3.077538000	1.524516000
C	2.521320000	-3.077538000	1.524516000
C	-1.206389000	4.060251000	3.457321000
C	-1.206389000	-4.060251000	3.457321000
C	-1.227710000	3.443146000	2.203625000
C	-1.227710000	-3.443146000	2.203625000
C	-2.521320000	3.077538000	1.524516000
C	-2.521320000	-3.077538000	1.524516000
H	0.000000000	1.284209000	-5.807531000
H	0.000000000	3.731253000	-5.286522000
H	0.000000000	4.215951000	-0.748344000
H	0.000000000	4.474097000	-2.932567000
H	0.000000000	4.847158000	5.060809000
H	0.000000000	-1.284209000	-5.807531000
H	0.000000000	-3.731253000	-5.286522000
H	0.000000000	-4.215951000	-0.748344000
H	0.000000000	-4.474097000	-2.932567000
H	0.000000000	-4.847158000	5.060809000
H	2.149945000	4.289846000	3.947701000
H	2.149945000	-4.289846000	3.947701000
H	2.523908000	2.014225000	1.255373000
H	2.523908000	-2.014225000	1.255373000
H	2.656952000	3.640815000	0.592485000
H	2.656952000	-3.640815000	0.592485000
H	3.374668000	3.279060000	2.178354000
H	3.374668000	-3.279060000	2.178354000
H	-2.149945000	4.289846000	3.947701000
H	-2.149945000	-4.289846000	3.947701000
H	-2.523908000	2.014225000	1.255373000
H	-2.523908000	-2.014225000	1.255373000
H	-2.656952000	3.640815000	0.592485000
H	-2.656952000	-3.640815000	0.592485000
H	-3.374668000	3.279060000	2.178354000
H	-3.374668000	-3.279060000	2.178354000
N	0.000000000	0.000000000	-1.511973000
N	0.000000000	2.464472000	0.345103000
N	0.000000000	-2.464472000	0.345103000

11. Ga(bicbz), C_{2v}, q = 0, S = 0

Ga	0.000000000	0.000000000	0.859259000
C	0.000000000	0.715461000	-3.717454000
C	0.000000000	1.108721000	-2.336883000
C	0.000000000	1.632303000	-4.764089000
C	0.000000000	2.506679000	-2.047365000
C	0.000000000	2.995511000	-4.470477000
C	0.000000000	3.142629000	-0.749383000
C	0.000000000	3.301166000	1.582401000
C	0.000000000	3.405101000	-3.139760000
C	0.000000000	4.628674000	4.030446000

C	0.000000000	-0.715461000	-3.717454000
C	0.000000000	-1.108721000	-2.336883000
C	0.000000000	-1.632303000	-4.764089000
C	0.000000000	-2.506679000	-2.047365000
C	0.000000000	-2.995511000	-4.470477000
C	0.000000000	-3.142629000	-0.749383000
C	0.000000000	-3.301166000	1.582401000
C	0.000000000	-3.405101000	-3.139760000
C	0.000000000	-4.628674000	4.030446000
C	1.206551000	4.289666000	3.418792000
C	1.206551000	-4.289666000	3.418792000
C	1.227594000	3.618054000	2.193298000
C	1.227594000	-3.618054000	2.193298000
C	2.519974000	3.227854000	1.525644000
C	2.519974000	-3.227854000	1.525644000
C	-1.206551000	4.289666000	3.418792000
C	-1.206551000	-4.289666000	3.418792000
C	-1.227594000	3.618054000	2.193298000
C	-1.227594000	-3.618054000	2.193298000
C	-2.519974000	3.227854000	1.525644000
C	-2.519974000	-3.227854000	1.525644000
H	0.000000000	1.285725000	-5.795218000
H	0.000000000	3.733860000	-5.267657000
H	0.000000000	4.242284000	-0.781512000
H	0.000000000	4.469877000	-2.917099000
H	0.000000000	5.149327000	4.985165000
H	0.000000000	-1.285725000	-5.795218000
H	0.000000000	-3.733860000	-5.267657000
H	0.000000000	-4.242284000	-0.781512000
H	0.000000000	-4.469877000	-2.917099000
H	0.000000000	-5.149327000	4.985165000
H	2.150094000	4.543798000	3.897269000
H	2.150094000	-4.543798000	3.897269000
H	2.525863000	2.156373000	1.289675000
H	2.525863000	-2.156373000	1.289675000
H	2.651348000	3.754977000	0.572506000
H	2.651348000	-3.754977000	0.572506000
H	3.375812000	3.453908000	2.168035000
H	3.375812000	-3.453908000	2.168035000
H	-2.150094000	4.543798000	3.897269000
H	-2.150094000	-4.543798000	3.897269000
H	-2.525863000	2.156373000	1.289675000
H	-2.525863000	-2.156373000	1.289675000
H	-2.651348000	3.754977000	0.572506000
H	-2.651348000	-3.754977000	0.572506000
H	-3.375812000	3.453908000	2.168035000
H	-3.375812000	-3.453908000	2.168035000
N	0.000000000	0.000000000	-1.506158000
N	0.000000000	2.553104000	0.388291000
N	0.000000000	-2.553104000	0.388291000

12. In(bicbz), C_{2v}, q = 0, S = 0

In	0.000000000	0.000000000	1.235573000
C	0.000000000	0.714563000	-3.661546000
C	0.000000000	1.106068000	-2.276598000
C	0.000000000	1.624305000	-4.712841000
C	0.000000000	2.509535000	-1.995257000
C	0.000000000	2.989050000	-4.426285000
C	0.000000000	3.183491000	-0.716709000
C	0.000000000	3.400897000	-3.097215000
C	0.000000000	3.513192000	1.583734000
C	0.000000000	5.064987000	3.900473000
C	0.000000000	-0.714563000	-3.661546000
C	0.000000000	-1.106068000	-2.276598000
C	0.000000000	-1.624305000	-4.712841000
C	0.000000000	-2.509535000	-1.995257000
C	0.000000000	-2.989050000	-4.426285000
C	0.000000000	-3.183491000	-0.716709000
C	0.000000000	-3.400897000	-3.097215000
C	0.000000000	-3.513192000	1.583734000
C	0.000000000	-5.064987000	3.900473000
C	1.206453000	4.672097000	3.321068000
C	1.206453000	-4.672097000	3.321068000
C	1.227372000	3.890822000	2.161620000
C	1.227372000	-3.890822000	2.161620000
C	2.522093000	3.452338000	1.528959000
C	2.522093000	-3.452338000	1.528959000
C	-1.206453000	4.672097000	3.321068000
C	-1.206453000	-4.672097000	3.321068000
C	-1.227372000	3.890822000	2.161620000
C	-1.227372000	-3.890822000	2.161620000
C	-2.522093000	3.452338000	1.528959000
C	-2.522093000	-3.452338000	1.528959000
H	0.000000000	1.271611000	-5.742232000
H	0.000000000	3.725363000	-5.225851000
H	0.000000000	4.280612000	-0.797171000
H	0.000000000	4.467325000	-2.881673000
H	0.000000000	5.671581000	4.803043000
H	0.000000000	-1.271611000	-5.742232000
H	0.000000000	-3.725363000	-5.225851000
H	0.000000000	-4.280612000	-0.797171000
H	0.000000000	-4.467325000	-2.881673000
H	0.000000000	-5.671581000	4.803043000
H	2.150557000	4.971971000	3.771681000
H	2.150557000	-4.971971000	3.771681000
H	2.544639000	2.362900000	1.400339000
H	2.544639000	-2.362900000	1.400339000
H	2.641347000	3.881943000	0.526626000
H	2.641347000	-3.881943000	0.526626000
H	3.377688000	3.753677000	2.140317000
H	3.377688000	-3.753677000	2.140317000

H	-2.150557000	4.971971000	3.771681000
H	-2.150557000	-4.971971000	3.771681000
H	-2.544639000	2.362900000	1.400339000
H	-2.544639000	-2.362900000	1.400339000
H	-2.641347000	3.881943000	0.526626000
H	-2.641347000	-3.881943000	0.526626000
H	-3.377688000	3.753677000	2.140317000
H	-3.377688000	-3.753677000	2.140317000
N	0.000000000	0.000000000	-1.446700000
N	0.000000000	2.662722000	0.456863000
N	0.000000000	-2.662722000	0.456863000

[M(dab)]$^-$

13. [Al(dab)]$^-$, C_{2v}, q = -1, S = 0

Al	0.000000000	0.000000000	-1.690632000
C	0.678417000	0.000000000	1.078960000
C	2.632805000	2.514950000	-0.086146000
C	2.632805000	-2.514950000	-0.086146000
C	2.694948000	0.000000000	-0.130268000
C	3.402664000	1.221056000	-0.070233000
C	3.402664000	-1.221056000	-0.070233000
C	4.797403000	1.205765000	0.039472000
C	4.797403000	-1.205765000	0.039472000
C	5.498645000	0.000000000	0.094671000
C	-0.678417000	0.000000000	1.078960000
C	-2.632805000	2.514950000	-0.086146000
C	-2.632805000	-2.514950000	-0.086146000
C	-2.694948000	0.000000000	-0.130268000
C	-3.402664000	1.221056000	-0.070233000
C	-3.402664000	-1.221056000	-0.070233000
C	-4.797403000	1.205765000	0.039472000
C	-4.797403000	-1.205765000	0.039472000
C	-5.498645000	0.000000000	0.094671000
H	1.284352000	0.000000000	1.981818000
H	1.914824000	2.540671000	0.742544000
H	1.914824000	-2.540671000	0.742544000
H	2.038326000	2.596120000	-1.003210000
H	2.038326000	-2.596120000	-1.003210000
H	3.308040000	3.375062000	-0.009549000
H	3.308040000	-3.375062000	-0.009549000
H	5.336795000	2.151072000	0.097505000
H	5.336795000	-2.151072000	0.097505000
H	6.583736000	0.000000000	0.189274000
H	-1.284352000	0.000000000	1.981818000
H	-1.914824000	2.540671000	0.742544000
H	-1.914824000	-2.540671000	0.742544000
H	-2.038326000	2.596120000	-1.003210000

H	-2.038326000	-2.596120000	-1.003210000
H	-3.308040000	3.375062000	-0.009549000
H	-3.308040000	-3.375062000	-0.009549000
H	-5.336795000	2.151072000	0.097505000
H	-5.336795000	-2.151072000	0.097505000
H	-6.583736000	0.000000000	0.189274000
N	1.286597000	0.000000000	-0.180087000
N	-1.286597000	0.000000000	-0.180087000

14. [Ga(dab)]$^-$, C_{2v}, q = -1, S = 0

Ga	0.000000000	0.000000000	-1.642879000
C	0.681162000	0.000000000	1.186885000
C	2.654284000	2.514755000	-0.043332000
C	2.654284000	-2.514755000	-0.043332000
C	2.711020000	0.000000000	-0.051460000
C	3.425317000	1.221346000	-0.053752000
C	3.425317000	-1.221346000	-0.053752000
C	4.824076000	1.205735000	-0.054478000
C	4.824076000	-1.205735000	-0.054478000
C	5.528297000	0.000000000	-0.053662000
C	-0.681162000	0.000000000	1.186885000
C	-2.654284000	2.514755000	-0.043332000
C	-2.654284000	-2.514755000	-0.043332000
C	-2.711020000	0.000000000	-0.051460000
C	-3.425317000	1.221346000	-0.053752000
C	-3.425317000	-1.221346000	-0.053752000
C	-4.824076000	1.205735000	-0.054478000
C	-4.824076000	-1.205735000	-0.054478000
C	-5.528297000	0.000000000	-0.053662000
H	1.269639000	0.000000000	2.102927000
H	1.970324000	2.547366000	0.812941000
H	1.970324000	-2.547366000	0.812941000
H	2.021719000	2.587364000	-0.935684000
H	2.021719000	-2.587364000	-0.935684000
H	3.331146000	3.376213000	-0.004144000
H	3.331146000	-3.376213000	-0.004144000
H	5.367009000	2.151152000	-0.048584000
H	5.367009000	-2.151152000	-0.048584000
H	6.617412000	0.000000000	-0.050045000
H	-1.269639000	0.000000000	2.102927000
H	-1.970324000	2.547366000	0.812941000
H	-1.970324000	-2.547366000	0.812941000
H	-2.021719000	2.587364000	-0.935684000
H	-2.021719000	-2.587364000	-0.935684000
H	-3.331146000	3.376213000	-0.004144000
H	-3.331146000	-3.376213000	-0.004144000
H	-5.367009000	2.151152000	-0.048584000
H	-5.367009000	-2.151152000	-0.048584000
H	-6.617412000	0.000000000	-0.050045000

N	1.312385000	0.000000000	-0.049321000
N	-1.312385000	0.000000000	-0.049321000

15. [In(dab)]$^{-}$, C_{2v}, q = -1, S = 0

In	0.000000000	0.000000000	-1.947910000
C	0.683195000	0.000000000	1.089589000
C	2.711681000	2.516361000	-0.044857000
C	2.711681000	-2.516361000	-0.044857000
C	2.762024000	0.000000000	-0.056846000
C	3.479558000	1.221087000	-0.035502000
C	3.479558000	-1.221087000	-0.035502000
C	4.877408000	1.205267000	-0.000585000
C	4.877408000	-1.205267000	-0.000585000
C	5.582154000	0.000000000	0.016293000
C	-0.683195000	0.000000000	1.089589000
C	-2.711681000	2.516361000	-0.044857000
C	-2.711681000	-2.516361000	-0.044857000
C	-2.762024000	0.000000000	-0.056846000
C	-3.479558000	1.221087000	-0.035502000
C	-3.479558000	-1.221087000	-0.035502000
C	-4.877408000	1.205267000	-0.000585000
C	-4.877408000	-1.205267000	-0.000585000
C	-5.582154000	0.000000000	0.016293000
H	1.243109000	0.000000000	2.025307000
H	1.980331000	2.536095000	0.771287000
H	1.980331000	-2.536095000	0.771287000
H	2.131403000	2.610306000	-0.970819000
H	2.131403000	-2.610306000	-0.970819000
H	3.387587000	3.374689000	0.047129000
H	3.387587000	-3.374689000	0.047129000
H	5.419555000	2.150923000	0.021300000
H	5.419555000	-2.150923000	0.021300000
H	6.670770000	0.000000000	0.047459000
H	-1.243109000	0.000000000	2.025307000
H	-1.980331000	2.536095000	0.771287000
H	-1.980331000	-2.536095000	0.771287000
H	-2.131403000	2.610306000	-0.970819000
H	-2.131403000	-2.610306000	-0.970819000
H	-3.387587000	3.374689000	0.047129000
H	-3.387587000	-3.374689000	0.047129000
H	-5.419555000	2.150923000	0.021300000
H	-5.419555000	-2.150923000	0.021300000
H	-6.670770000	0.000000000	0.047459000
N	1.367701000	0.000000000	-0.113935000
N	-1.367701000	0.000000000	-0.113935000

Aryltrielylenes

16. Al(Ph^{Fl}), C_{2v}, q = 0, S = 0

Al	0.000000000	0.000000000	-1.488504000
C	0.000000000	0.000000000	0.591704000
C	0.000000000	0.000000000	3.467494000
C	0.000000000	1.184653000	1.357013000
C	0.000000000	1.197130000	2.754015000
C	0.000000000	2.591399000	3.341212000
C	0.000000000	2.616189000	0.806467000
C	0.000000000	3.512837000	2.086050000
C	0.000000000	-1.184653000	1.357013000
C	0.000000000	-1.197130000	2.754015000
C	0.000000000	-2.591399000	3.341212000
C	0.000000000	-2.616189000	0.806467000
C	0.000000000	-3.512837000	2.086050000
C	0.731487000	3.231522000	-1.413216000
C	0.731487000	-3.231522000	-1.413216000
C	1.178338000	2.893533000	-0.119822000
C	1.178338000	-2.893533000	-0.119822000
C	1.254868000	2.826227000	4.198616000
C	1.254868000	-2.826227000	4.198616000
C	1.659173000	3.527426000	-2.408660000
C	1.659173000	-3.527426000	-2.408660000
C	2.528265000	2.875859000	0.178588000
C	2.528265000	-2.875859000	0.178588000
C	3.020074000	3.517961000	-2.092284000
C	3.020074000	-3.517961000	-2.092284000
C	3.479192000	3.210639000	-0.802066000
C	3.479192000	-3.210639000	-0.802066000
C	4.962766000	3.239408000	-0.422650000
C	4.962766000	-3.239408000	-0.422650000
C	5.174042000	4.243679000	0.729631000
C	5.174042000	-4.243679000	0.729631000
C	5.394322000	1.834519000	0.037172000
C	5.394322000	-1.834519000	0.037172000
C	5.864199000	3.657358000	-1.592497000
C	5.864199000	-3.657358000	-1.592497000
C	-0.731487000	3.231522000	-1.413216000
C	-0.731487000	-3.231522000	-1.413216000
C	-1.178338000	2.893533000	-0.119822000
C	-1.178338000	-2.893533000	-0.119822000
C	-1.254868000	2.826227000	4.198616000
C	-1.254868000	-2.826227000	4.198616000
C	-1.659173000	3.527426000	-2.408660000
C	-1.659173000	-3.527426000	-2.408660000
C	-2.528265000	2.875859000	0.178588000
C	-2.528265000	-2.875859000	0.178588000

C	-3.020074000	3.517961000	-2.092284000
C	-3.020074000	-3.517961000	-2.092284000
C	-3.479192000	3.210639000	-0.802066000
C	-3.479192000	-3.210639000	-0.802066000
C	-4.962766000	3.239408000	-0.422650000
C	-4.962766000	-3.239408000	-0.422650000
C	-5.174042000	4.243679000	0.729631000
C	-5.174042000	-4.243679000	0.729631000
C	-5.394322000	1.834519000	0.037172000
C	-5.394322000	-1.834519000	0.037172000
C	-5.864199000	3.657358000	-1.592497000
C	-5.864199000	-3.657358000	-1.592497000
H	0.000000000	0.000000000	4.554718000
H	0.880516000	4.157085000	2.082741000
H	0.880516000	-4.157085000	2.082741000
H	1.270596000	2.143041000	5.054355000
H	1.270596000	-2.143041000	5.054355000
H	1.271136000	3.855458000	4.575654000
H	1.271136000	-3.855458000	4.575654000
H	1.334109000	3.774029000	-3.416289000
H	1.334109000	-3.774029000	-3.416289000
H	2.159801000	2.656226000	3.606965000
H	2.159801000	-2.656226000	3.606965000
H	2.847051000	2.615292000	1.182186000
H	2.847051000	-2.615292000	1.182186000
H	3.730589000	3.765340000	-2.872282000
H	3.730589000	-3.765340000	-2.872282000
H	4.595173000	3.961940000	1.613280000
H	4.595173000	-3.961940000	1.613280000
H	4.800963000	1.491615000	0.888601000
H	4.800963000	-1.491615000	0.888601000
H	4.865764000	5.248406000	0.422043000
H	4.865764000	-5.248406000	0.422043000
H	5.264625000	1.118816000	-0.777563000
H	5.264625000	-1.118816000	-0.777563000
H	5.614620000	4.662297000	-1.947476000
H	5.614620000	-4.662297000	-1.947476000
H	5.778382000	2.957493000	-2.429625000
H	5.778382000	-2.957493000	-2.429625000
H	6.233549000	4.272110000	1.008226000
H	6.233549000	-4.272110000	1.008226000
H	6.449849000	1.841466000	0.331799000
H	6.449849000	-1.841466000	0.331799000
H	6.907240000	3.662292000	-1.260206000
H	6.907240000	-3.662292000	-1.260206000
H	-0.880516000	4.157085000	2.082741000
H	-0.880516000	-4.157085000	2.082741000
H	-1.270596000	2.143041000	5.054355000
H	-1.270596000	-2.143041000	5.054355000
H	-1.271136000	3.855458000	4.575654000
H	-1.271136000	-3.855458000	4.575654000

H	-1.334109000	3.774029000	-3.416289000
H	-1.334109000	-3.774029000	-3.416289000
H	-2.159801000	2.656226000	3.606965000
H	-2.159801000	-2.656226000	3.606965000
H	-2.847051000	2.615292000	1.182186000
H	-2.847051000	-2.615292000	1.182186000
H	-3.730589000	3.765340000	-2.872282000
H	-3.730589000	-3.765340000	-2.872282000
H	-4.595173000	3.961940000	1.613280000
H	-4.595173000	-3.961940000	1.613280000
H	-4.800963000	1.491615000	0.888601000
H	-4.800963000	-1.491615000	0.888601000
H	-4.865764000	5.248406000	0.422043000
H	-4.865764000	-5.248406000	0.422043000
H	-5.264625000	1.118816000	-0.777563000
H	-5.264625000	-1.118816000	-0.777563000
H	-5.614620000	4.662297000	-1.947476000
H	-5.614620000	-4.662297000	-1.947476000
H	-5.778382000	2.957493000	-2.429625000
H	-5.778382000	-2.957493000	-2.429625000
H	-6.233549000	4.272110000	1.008226000
H	-6.233549000	-4.272110000	1.008226000
H	-6.449849000	1.841466000	0.331799000
H	-6.449849000	-1.841466000	0.331799000
H	-6.907240000	3.662292000	-1.260206000
H	-6.907240000	-3.662292000	-1.260206000

17. Ga(Ph^{Fl}), C_{2v}, q = 0, S = 0

Ga	0.000000000	0.000000000	-1.546224000
C	0.000000000	0.000000000	0.600358000
C	0.000000000	0.000000000	3.473770000
C	0.000000000	1.182593000	1.361729000
C	0.000000000	1.196632000	2.759325000
C	0.000000000	2.591744000	3.344392000
C	0.000000000	2.613289000	0.810316000
C	0.000000000	3.511592000	2.087854000
C	0.000000000	-1.182593000	1.361729000
C	0.000000000	-1.196632000	2.759325000
C	0.000000000	-2.591744000	3.344392000
C	0.000000000	-2.613289000	0.810316000
C	0.000000000	-3.511592000	2.087854000
C	0.731489000	3.205785000	-1.414045000
C	0.731489000	-3.205785000	-1.414045000
C	1.177849000	2.884172000	-0.116409000
C	1.177849000	-2.884172000	-0.116409000
C	1.254839000	2.828527000	4.201262000
C	1.254839000	-2.828527000	4.201262000
C	1.659993000	3.481676000	-2.414733000
C	1.659993000	-3.481676000	-2.414733000

C	2.527779000	2.866687000	0.182173000
C	2.527779000	-2.866687000	0.182173000
C	3.021205000	3.474692000	-2.098407000
C	3.021205000	-3.474692000	-2.098407000
C	3.479480000	3.187337000	-0.803091000
C	3.479480000	-3.187337000	-0.803091000
C	4.962356000	3.227378000	-0.422248000
C	4.962356000	-3.227378000	-0.422248000
C	5.167916000	4.254829000	0.710609000
C	5.167916000	-4.254829000	0.710609000
C	5.398604000	1.833496000	0.064381000
C	5.398604000	-1.833496000	0.064381000
C	5.864460000	3.625128000	-1.598559000
C	5.864460000	-3.625128000	-1.598559000
C	-0.731489000	3.205785000	-1.414045000
C	-0.731489000	-3.205785000	-1.414045000
C	-1.177849000	2.884172000	-0.116409000
C	-1.177849000	-2.884172000	-0.116409000
C	-1.254839000	2.828527000	4.201262000
C	-1.254839000	-2.828527000	4.201262000
C	-1.659993000	3.481676000	-2.414733000
C	-1.659993000	-3.481676000	-2.414733000
C	-2.527779000	2.866687000	0.182173000
C	-2.527779000	-2.866687000	0.182173000
C	-3.021205000	3.474692000	-2.098407000
C	-3.021205000	-3.474692000	-2.098407000
C	-3.479480000	3.187337000	-0.803091000
C	-3.479480000	-3.187337000	-0.803091000
C	-4.962356000	3.227378000	-0.422248000
C	-4.962356000	-3.227378000	-0.422248000
C	-5.167916000	4.254829000	0.710609000
C	-5.167916000	-4.254829000	0.710609000
C	-5.398604000	1.833496000	0.064381000
C	-5.398604000	-1.833496000	0.064381000
C	-5.864460000	3.625128000	-1.598559000
C	-5.864460000	-3.625128000	-1.598559000
H	0.000000000	0.000000000	4.560938000
H	0.880453000	4.156010000	2.083926000
H	0.880453000	-4.156010000	2.083926000
H	1.270970000	2.146390000	5.057818000
H	1.270970000	-2.146390000	5.057818000
H	1.271062000	3.858414000	4.576650000
H	1.271062000	-3.858414000	4.576650000
H	1.335682000	3.714295000	-3.426054000
H	1.335682000	-3.714295000	-3.426054000
H	2.159675000	2.657648000	3.609669000
H	2.159675000	-2.657648000	3.609669000
H	2.845857000	2.618610000	1.189098000
H	2.845857000	-2.618610000	1.189098000
H	3.732161000	3.709504000	-2.881936000
H	3.732161000	-3.709504000	-2.881936000

H	4.594108000	3.984283000	1.601023000
H	4.594108000	-3.984283000	1.601023000
H	4.803409000	1.502104000	0.919031000
H	4.803409000	-1.502104000	0.919031000
H	4.849356000	5.250615000	0.385215000
H	4.849356000	-5.250615000	0.385215000
H	5.276136000	1.103428000	-0.738475000
H	5.276136000	-1.103428000	-0.738475000
H	5.613207000	4.622413000	-1.973141000
H	5.613207000	-4.622413000	-1.973141000
H	5.781606000	2.909227000	-2.422444000
H	5.781606000	-2.909227000	-2.422444000
H	6.227919000	4.298213000	0.985252000
H	6.227919000	-4.298213000	0.985252000
H	6.452860000	1.850704000	0.363026000
H	6.452860000	-1.850704000	0.363026000
H	6.906927000	3.638899000	-1.264658000
H	6.906927000	-3.638899000	-1.264658000
H	-0.880453000	4.156010000	2.083926000
H	-0.880453000	-4.156010000	2.083926000
H	-1.270970000	2.146390000	5.057818000
H	-1.270970000	-2.146390000	5.057818000
H	-1.271062000	3.858414000	4.576650000
H	-1.271062000	-3.858414000	4.576650000
H	-1.335682000	3.714295000	-3.426054000
H	-1.335682000	-3.714295000	-3.426054000
H	-2.159675000	2.657648000	3.609669000
H	-2.159675000	-2.657648000	3.609669000
H	-2.845857000	2.618610000	1.189098000
H	-2.845857000	-2.618610000	1.189098000
H	-3.732161000	3.709504000	-2.881936000
H	-3.732161000	-3.709504000	-2.881936000
H	-4.594108000	3.984283000	1.601023000
H	-4.594108000	-3.984283000	1.601023000
H	-4.803409000	1.502104000	0.919031000
H	-4.803409000	-1.502104000	0.919031000
H	-4.849356000	5.250615000	0.385215000
H	-4.849356000	-5.250615000	0.385215000
H	-5.276136000	1.103428000	-0.738475000
H	-5.276136000	-1.103428000	-0.738475000
H	-5.613207000	4.622413000	-1.973141000
H	-5.613207000	-4.622413000	-1.973141000
H	-5.781606000	2.909227000	-2.422444000
H	-5.781606000	-2.909227000	-2.422444000
H	-6.227919000	4.298213000	0.985252000
H	-6.227919000	-4.298213000	0.985252000
H	-6.452860000	1.850704000	0.363026000
H	-6.452860000	-1.850704000	0.363026000
H	-6.906927000	3.638899000	-1.264658000
H	-6.906927000	-3.638899000	-1.264658000

18. In(Ph^{Fl}), C_{2v}, q = 0, S = 0

In	0.000000000	0.000000000	-1.837028000
C	0.000000000	0.000000000	0.569764000
C	0.000000000	0.000000000	3.450160000
C	0.000000000	1.181554000	1.335105000
C	0.000000000	1.194331000	2.734909000
C	0.000000000	2.584246000	3.330153000
C	0.000000000	2.618494000	0.795374000
C	0.000000000	3.511393000	2.080998000
C	0.000000000	-1.181554000	1.335105000
C	0.000000000	-1.194331000	2.734909000
C	0.000000000	-2.584246000	3.330153000
C	0.000000000	-2.618494000	0.795374000
C	0.000000000	-3.511393000	2.080998000
C	0.731834000	3.326910000	-1.395822000
C	0.731834000	-3.326910000	-1.395822000
C	1.177505000	2.920669000	-0.121189000
C	1.177505000	-2.920669000	-0.121189000
C	1.254385000	2.814705000	4.190018000
C	1.254385000	-2.814705000	4.190018000
C	1.661834000	3.653256000	-2.380429000
C	1.661834000	-3.653256000	-2.380429000
C	2.528076000	2.868874000	0.171142000
C	2.528076000	-2.868874000	0.171142000
C	3.024013000	3.610661000	-2.068867000
C	3.024013000	-3.610661000	-2.068867000
C	3.481928000	3.238977000	-0.795004000
C	3.481928000	-3.238977000	-0.795004000
C	4.966799000	3.233894000	-0.418285000
C	4.966799000	-3.233894000	-0.418285000
C	5.188329000	4.164282000	0.793491000
C	5.188329000	-4.164282000	0.793491000
C	5.387621000	1.800532000	-0.045470000
C	5.387621000	-1.800532000	-0.045470000
C	5.869504000	3.716997000	-1.562127000
C	5.869504000	-3.716997000	-1.562127000
C	-0.731834000	3.326910000	-1.395822000
C	-0.731834000	-3.326910000	-1.395822000
C	-1.177505000	2.920669000	-0.121189000
C	-1.177505000	-2.920669000	-0.121189000
C	-1.254385000	2.814705000	4.190018000
C	-1.254385000	-2.814705000	4.190018000
C	-1.661834000	3.653256000	-2.380429000
C	-1.661834000	-3.653256000	-2.380429000
C	-2.528076000	2.868874000	0.171142000
C	-2.528076000	-2.868874000	0.171142000
C	-3.024013000	3.610661000	-2.068867000
C	-3.024013000	-3.610661000	-2.068867000

C	-3.481928000	3.238977000	-0.795004000
C	-3.481928000	-3.238977000	-0.795004000
C	-4.966799000	3.233894000	-0.418285000
C	-4.966799000	-3.233894000	-0.418285000
C	-5.188329000	4.164282000	0.793491000
C	-5.188329000	-4.164282000	0.793491000
C	-5.387621000	1.800532000	-0.045470000
C	-5.387621000	-1.800532000	-0.045470000
C	-5.869504000	3.716997000	-1.562127000
C	-5.869504000	-3.716997000	-1.562127000
H	0.000000000	0.000000000	4.537269000
H	0.880548000	4.155633000	2.080461000
H	0.880548000	-4.155633000	2.080461000
H	1.268916000	2.126486000	5.041870000
H	1.268916000	-2.126486000	5.041870000
H	1.271250000	3.841887000	4.573278000
H	1.271250000	-3.841887000	4.573278000
H	1.338623000	3.950662000	-3.375105000
H	1.338623000	-3.950662000	-3.375105000
H	2.159642000	2.647192000	3.598134000
H	2.159642000	-2.647192000	3.598134000
H	2.843813000	2.553484000	1.159806000
H	2.843813000	-2.553484000	1.159806000
H	3.735794000	3.885637000	-2.838667000
H	3.735794000	-3.885637000	-2.838667000
H	4.615053000	3.829795000	1.662079000
H	4.615053000	-3.829795000	1.662079000
H	4.788219000	1.410180000	0.780580000
H	4.788219000	-1.410180000	0.780580000
H	4.880847000	5.187062000	0.551377000
H	4.880847000	-5.187062000	0.551377000
H	5.256635000	1.136369000	-0.903041000
H	5.256635000	-1.136369000	-0.903041000
H	5.622195000	4.741393000	-1.858517000
H	5.622195000	-4.741393000	-1.858517000
H	5.783528000	3.067092000	-2.438720000
H	5.783528000	-3.067092000	-2.438720000
H	6.249807000	4.172811000	1.065975000
H	6.249807000	-4.172811000	1.065975000
H	6.441876000	1.780948000	0.253051000
H	6.441876000	-1.780948000	0.253051000
H	6.912390000	3.700591000	-1.229379000
H	6.912390000	-3.700591000	-1.229379000
H	-0.880548000	4.155633000	2.080461000
H	-0.880548000	-4.155633000	2.080461000
H	-1.268916000	2.126486000	5.041870000
H	-1.268916000	-2.126486000	5.041870000
H	-1.271250000	3.841887000	4.573278000
H	-1.271250000	-3.841887000	4.573278000
H	-1.338623000	3.950662000	-3.375105000
H	-1.338623000	-3.950662000	-3.375105000

H	-2.159642000	2.647192000	3.598134000
H	-2.159642000	-2.647192000	3.598134000
H	-2.843813000	2.553484000	1.159806000
H	-2.843813000	-2.553484000	1.159806000
H	-3.735794000	3.885637000	-2.838667000
H	-3.735794000	-3.885637000	-2.838667000
H	-4.615053000	3.829795000	1.662079000
H	-4.615053000	-3.829795000	1.662079000
H	-4.788219000	1.410180000	0.780580000
H	-4.788219000	-1.410180000	0.780580000
H	-4.880847000	5.187062000	0.551377000
H	-4.880847000	-5.187062000	0.551377000
H	-5.256635000	1.136369000	-0.903041000
H	-5.256635000	-1.136369000	-0.903041000
H	-5.622195000	4.741393000	-1.858517000
H	-5.622195000	-4.741393000	-1.858517000
H	-5.783528000	3.067092000	-2.438720000
H	-5.783528000	-3.067092000	-2.438720000
H	-6.249807000	4.172811000	1.065975000
H	-6.249807000	-4.172811000	1.065975000
H	-6.441876000	1.780948000	0.253051000
H	-6.441876000	-1.780948000	0.253051000
H	-6.912390000	3.700591000	-1.229379000
H	-6.912390000	-3.700591000	-1.229379000

Hydrotrispyrazolylborates

19. Al(TpMe), C_{3v}, q = 0, S = 0

Al	0.000000000	0.000000000	2.053259000
B	0.000000000	0.000000000	-1.198915000
C	1.321088000	2.288192000	-1.281470000
C	1.321088000	-2.288192000	-1.281470000
C	1.406738000	2.436542000	-2.765551000
C	1.406738000	-2.436542000	-2.765551000
C	1.475812000	2.556182000	0.928741000
C	1.475812000	-2.556182000	0.928741000
C	1.767864000	3.062030000	2.302878000
C	1.767864000	-3.062030000	2.302878000
C	1.813923000	3.141806000	-0.298887000
C	1.813923000	-3.141806000	-0.298887000
C	-2.642177000	0.000000000	-1.281470000
C	-2.813476000	0.000000000	-2.765551000
C	-2.951625000	0.000000000	0.928741000
C	-3.535728000	0.000000000	2.302878000
C	-3.627845000	0.000000000	-0.298887000
H	0.000000000	0.000000000	-2.386671000
H	0.924219000	3.369182000	-3.082809000
H	0.924219000	-3.369182000	-3.082809000
H	0.927706000	1.606833000	-3.285765000

H	0.927706000	-1.606833000	-3.285765000
H	1.316232000	4.051248000	2.448533000
H	1.316232000	-4.051248000	2.448533000
H	1.373584000	2.379117000	3.060184000
H	1.373584000	-2.379117000	3.060184000
H	2.348657000	4.067994000	-0.455774000
H	2.348657000	-4.067994000	-0.455774000
H	2.455687000	2.484988000	-3.082809000
H	2.455687000	-2.484988000	-3.082809000
H	2.850368000	3.165515000	2.448533000
H	2.850368000	-3.165515000	2.448533000
H	-1.855411000	0.000000000	-3.285765000
H	-2.747168000	0.000000000	3.060184000
H	-3.379907000	0.884194000	-3.082809000
H	-3.379907000	-0.884194000	-3.082809000
H	-4.166600000	0.885733000	2.448533000
H	-4.166600000	-0.885733000	2.448533000
H	-4.697315000	0.000000000	-0.455774000
N	0.721508000	1.249688000	-0.652079000
N	0.721508000	-1.249688000	-0.652079000
N	0.812028000	1.406474000	0.705725000
N	0.812028000	-1.406474000	0.705725000
N	-1.443016000	0.000000000	-0.652079000
N	-1.624057000	0.000000000	0.705725000

20. Ga(TpMe), C_{3v}, q = 0, *S* = 0

Ga	0.000000000	0.000000000	2.331446000
B	0.000000000	0.000000000	-1.091565000
C	1.314810000	2.277318000	-1.273346000
C	1.314810000	-2.277318000	-1.273346000
C	1.370458000	2.373702000	-2.763951000
C	1.370458000	-2.373702000	-2.763951000
C	1.520792000	2.634089000	0.916864000
C	1.520792000	-2.634089000	0.916864000
C	1.831360000	3.172008000	-0.341853000
C	1.831360000	-3.172008000	-0.341853000
C	1.839974000	3.186929000	2.268237000
C	1.839974000	-3.186929000	2.268237000
C	-2.629620000	0.000000000	-1.273346000
C	-2.740915000	0.000000000	-2.763951000
C	-3.041584000	0.000000000	0.916864000
C	-3.662719000	0.000000000	-0.341853000
C	-3.679949000	0.000000000	2.268237000
H	0.000000000	0.000000000	-2.278001000
H	0.881645000	3.295347000	-3.103384000
H	0.881645000	-3.295347000	-3.103384000
H	0.882392000	1.528348000	-3.247972000
H	0.882392000	-1.528348000	-3.247972000
H	1.391818000	4.180317000	2.395223000

H	1.391818000	-4.180317000	2.395223000
H	1.463351000	2.534598000	3.060137000
H	1.463351000	-2.534598000	3.060137000
H	2.361386000	4.090041000	-0.553956000
H	2.361386000	-4.090041000	-0.553956000
H	2.413032000	2.411201000	-3.103384000
H	2.413032000	-2.411201000	-3.103384000
H	2.924351000	3.295508000	2.395223000
H	2.924351000	-3.295508000	2.395223000
H	-1.764784000	0.000000000	-3.247972000
H	-2.926702000	0.000000000	3.060137000
H	-3.294677000	0.884147000	-3.103384000
H	-3.294677000	-0.884147000	-3.103384000
H	-4.316169000	0.884808000	2.395223000
H	-4.316169000	-0.884808000	2.395223000
H	-4.722772000	0.000000000	-0.553956000
N	0.729628000	1.263753000	-0.581493000
N	0.729628000	-1.263753000	-0.581493000
N	0.855230000	1.481302000	0.758370000
N	0.855230000	-1.481302000	0.758370000
N	-1.459256000	0.000000000	-0.581493000
N	-1.710460000	0.000000000	0.758370000

21. In(TpMe), C_{3v}, q = 0, S = 0

In	0.000000000	0.000000000	2.618940000
B	0.000000000	0.000000000	-0.996184000
C	1.305402000	2.261023000	-1.260014000
C	1.305402000	-2.261023000	-1.260014000
C	1.335047000	2.312369000	-2.754209000
C	1.335047000	-2.312369000	-2.754209000
C	1.559725000	2.701523000	0.907414000
C	1.559725000	-2.701523000	0.907414000
C	1.842343000	3.191032000	-0.376976000
C	1.842343000	-3.191032000	-0.376976000
C	1.913421000	3.314142000	2.224159000
C	1.913421000	-3.314142000	2.224159000
C	-2.610805000	0.000000000	-1.260014000
C	-2.670094000	0.000000000	-2.754209000
C	-3.119450000	0.000000000	0.907414000
C	-3.684686000	0.000000000	-0.376976000
C	-3.826841000	0.000000000	2.224159000
H	0.000000000	0.000000000	-2.181561000
H	0.840103000	1.455101000	-3.208535000
H	0.840103000	-1.455101000	-3.208535000
H	0.841028000	3.224638000	-3.111381000
H	0.841028000	-3.224638000	-3.111381000
H	1.468575000	4.312839000	2.318763000
H	1.468575000	-4.312839000	2.318763000
H	1.559752000	2.701570000	3.057608000

H	1.559752000	-2.701570000	3.057608000
H	2.367127000	4.099984000	-0.637386000
H	2.367127000	-4.099984000	-0.637386000
H	2.372104000	2.340670000	-3.111381000
H	2.372104000	-2.340670000	-3.111381000
H	3.000741000	3.428243000	2.318763000
H	3.000741000	-3.428243000	2.318763000
H	-1.680205000	0.000000000	-3.208535000
H	-3.119504000	0.000000000	3.057608000
H	-3.213132000	0.883967000	-3.111381000
H	-3.213132000	-0.883967000	-3.111381000
H	-4.469316000	0.884596000	2.318763000
H	-4.469316000	-0.884596000	2.318763000
H	-4.734254000	0.000000000	-0.637386000
N	0.735285000	1.273551000	-0.515547000
N	0.735285000	-1.273551000	-0.515547000
N	0.891060000	1.543362000	0.813664000
N	0.891060000	-1.543362000	0.813664000
N	-1.470570000	0.000000000	-0.515547000
N	-1.782121000	0.000000000	0.813664000

22. Al(TpCF$_3$), C_{3v}, q = 0, S = 0

Al	0.000000000	0.000000000	2.399180000
B	0.000000000	0.000000000	-1.070799000
C	1.316726000	2.280636000	-1.244509000
C	1.316726000	-2.280636000	-1.244509000
C	1.432691000	2.481493000	-2.735513000
C	1.432691000	-2.481493000	-2.735513000
C	1.523060000	2.638017000	0.911071000
C	1.523060000	-2.638017000	0.911071000
C	1.837630000	3.182869000	-0.333536000
C	1.837630000	-3.182869000	-0.333536000
C	1.883821000	3.262873000	2.226328000
C	1.883821000	-3.262873000	2.226328000
C	-2.633452000	0.000000000	-1.244509000
C	-2.865382000	0.000000000	-2.735513000
C	-3.046119000	0.000000000	0.911071000
C	-3.675260000	0.000000000	-0.333536000
C	-3.767641000	0.000000000	2.226328000
F	0.853395000	3.661353000	-3.075028000
F	0.853395000	-3.661353000	-3.075028000
F	0.882533000	1.528592000	-3.498075000
F	0.882533000	-1.528592000	-3.498075000
F	1.339777000	4.501497000	2.322712000
F	1.339777000	-4.501497000	2.322712000
F	1.469318000	2.544933000	3.285086000
F	1.469318000	-2.544933000	3.285086000
F	2.744127000	2.569738000	-3.075028000
F	2.744127000	-2.569738000	-3.075028000

F	3.228522000	3.411030000	2.322712000
F	3.228522000	-3.411030000	2.322712000
F	-1.765066000	0.000000000	-3.498075000
F	-2.938635000	0.000000000	3.285086000
F	-3.597522000	1.091615000	-3.075028000
F	-3.597522000	-1.091615000	-3.075028000
F	-4.568299000	1.090467000	2.322712000
F	-4.568299000	-1.090467000	2.322712000
H	0.000000000	0.000000000	-2.230699000
H	2.366327000	4.098599000	-0.547355000
H	2.366327000	-4.098599000	-0.547355000
H	-4.732654000	0.000000000	-0.547355000
N	0.729910000	1.264241000	-0.555748000
N	0.729910000	-1.264241000	-0.555748000
N	0.855868000	1.482406000	0.776331000
N	0.855868000	-1.482406000	0.776331000
N	-1.459820000	0.000000000	-0.555748000
N	-1.711735000	0.000000000	0.776331000

23. Ga(TpCF$_3$), C_{3v}, q = 0, S = 0

Ga	0.000000000	0.000000000	2.634029000
B	0.000000000	0.000000000	-1.021466000
C	1.316876000	2.280896000	-1.248050000
C	1.316876000	-2.280896000	-1.248050000
C	1.420590000	2.460534000	-2.743495000
C	1.420590000	-2.460534000	-2.743495000
C	1.547530000	2.680401000	0.897343000
C	1.547530000	-2.680401000	0.897343000
C	1.849294000	3.203072000	-0.362419000
C	1.849294000	-3.203072000	-0.362419000
C	1.905760000	3.300873000	2.213084000
C	1.905760000	-3.300873000	2.213084000
C	-2.633752000	0.000000000	-1.248050000
C	-2.841179000	0.000000000	-2.743495000
C	-3.095060000	0.000000000	0.897343000
C	-3.698589000	0.000000000	-0.362419000
C	-3.811520000	0.000000000	2.213084000
F	0.838402000	3.638001000	-3.095070000
F	0.838402000	-3.638001000	-3.095070000
F	0.867019000	1.501721000	-3.495240000
F	0.867019000	-1.501721000	-3.495240000
F	1.360486000	4.535856000	2.332283000
F	1.360486000	-4.535856000	2.332283000
F	1.480512000	2.564322000	3.267866000
F	1.480512000	-2.564322000	3.267866000
F	2.731400000	2.545078000	-3.095070000
F	2.731400000	-2.545078000	-3.095070000
F	3.247924000	3.446143000	2.332283000
F	3.247924000	-3.446143000	2.332283000

F	-1.734039000	0.000000000	-3.495240000
F	-2.961023000	0.000000000	3.267866000
F	-3.569802000	1.092923000	-3.095070000
F	-3.569802000	-1.092923000	-3.095070000
F	-4.608410000	1.089713000	2.332283000
F	-4.608410000	-1.089713000	2.332283000
H	0.000000000	0.000000000	-2.182179000
H	2.375025000	4.113665000	-0.604327000
H	2.375025000	-4.113665000	-0.604327000
H	-4.750051000	0.000000000	-0.604327000
N	0.736564000	1.275766000	-0.525671000
N	0.736564000	-1.275766000	-0.525671000
N	0.880819000	1.525624000	0.795031000
N	0.880819000	-1.525624000	0.795031000
N	-1.473128000	0.000000000	-0.525671000
N	-1.761639000	0.000000000	0.795031000

24. In(TpCF$_3$), C_{3v}, q = 0, S = 0

In	0.000000000	0.000000000	2.948992000
B	0.000000000	0.000000000	-0.961580000
C	1.314452000	2.276698000	-1.242040000
C	1.314452000	-2.276698000	-1.242040000
C	1.406670000	2.436423000	-2.742808000
C	1.406670000	-2.436423000	-2.742808000
C	1.573364000	2.725146000	0.890753000
C	1.573364000	-2.725146000	0.890753000
C	1.858152000	3.218413000	-0.385252000
C	1.858152000	-3.218413000	-0.385252000
C	1.954454000	3.385213000	2.181803000
C	1.954454000	-3.385213000	2.181803000
C	-2.628904000	0.000000000	-1.242040000
C	-2.813339000	0.000000000	-2.742808000
C	-3.146728000	0.000000000	0.890753000
C	-3.716303000	0.000000000	-0.385252000
C	-3.908908000	0.000000000	2.181803000
F	0.822286000	3.610799000	-3.108683000
F	0.822286000	-3.610799000	-3.108683000
F	0.847957000	1.468704000	-3.478021000
F	0.847957000	-1.468704000	-3.478021000
F	1.411468000	4.623362000	2.279802000
F	1.411468000	-4.623362000	2.279802000
F	1.549330000	2.683519000	3.272511000
F	1.549330000	-2.683519000	3.272511000
F	2.715900000	2.517520000	-3.108683000
F	2.715900000	-2.517520000	-3.108683000
F	3.298215000	3.534048000	2.279802000
F	3.298215000	-3.534048000	2.279802000
F	-1.695914000	0.000000000	-3.478021000
F	-3.098661000	0.000000000	3.272511000

F	-3.538187000	1.093279000	-3.108683000
F	-3.538187000	-1.093279000	-3.108683000
F	-4.709682000	1.089314000	2.279802000
F	-4.709682000	-1.089314000	2.279802000
H	0.000000000	0.000000000	-2.122505000
H	2.380308000	4.122815000	-0.656852000
H	2.380308000	-4.122815000	-0.656852000
H	-4.760616000	0.000000000	-0.656852000
N	0.742291000	1.285686000	-0.486298000
N	0.742291000	-1.285686000	-0.486298000
N	0.904982000	1.567474000	0.827674000
N	0.904982000	-1.567474000	0.827674000
N	-1.484582000	0.000000000	-0.486298000
N	-1.809963000	0.000000000	0.827674000
